# Systematic Review and Meta-Analysis of Laparoscopic versus Robotic-Assisted Surgery for Colon Cancer: Efficacy, Safety, and Outcomes—A Focus on Studies from 2020–2024

**DOI:** 10.3390/cancers16081552

**Published:** 2024-04-18

**Authors:** Roxana Loriana Negrut, Adrian Cote, Vasile Aurel Caus, Adrian Marius Maghiar

**Affiliations:** 1 Department of Medicine, Faculty of Medicine and Pharmacy, Doctoral School of Biomedical Sciences, University of Oradea, 410087 Oradea, Romania; popa.roxanaloriana@student.uoradea.ro (R.L.N.);; 2 County Clinical Emergency Hospital Bihor, 410087 Oradea, Romania; 3 Department of Surgical Disciplines, Faculty of Medicine and Pharmacy, University of Oradea, 410073 Oradea, Romania; 4 Department of Mathematics and Computer Science, University of Oradea, 410087 Oradea, Romania

**Keywords:** colon cancer, surgery, laparoscopic surgery, robotic surgery, outcomes

## Abstract

**Simple Summary:**

In this research, we explored the latest advancements in minimally invasive surgery for colon cancer, by comparing laparoscopic surgery to the robotic approach. Our goal was to determine which method has better outcomes in terms of length of surgery, hospital stay, the likelihood of conversion, rate of complications, anastomotic leaks, and the effectiveness of tumor removal by evaluating the number of lymphatic nodes harvested. The findings could help surgeons and patients make more informed decisions related to the surgical options, considering the benefits of each technique. This summary aims to give a straightforward overview of the importance of this research and how it could impact the surgical approach.

**Abstract:**

Background: Minimally invasive surgery in the treatment of colon cancer has significantly advanced over the years. This systematic review and meta-analysis aimed to compare the operative outcomes of robotic and laparoscopic surgery in the treatment of colon cancer, focusing on operative time, hospital stay, conversion rates, anastomotic leak rates, and total number lymph node harvested. Methods: Following PRISMA guidelines, we conducted a systematic search across four databases up to January 2024, registering our protocol with PROSPERO (CRD42024513326). We included studies comparing robotic and laparoscopic surgeries for colon cancer, assessing operative time, hospital length of stay, and other perioperative outcomes. Risk of bias was evaluated using the JBI Critical Appraisal Checklist. Statistical analysis utilized a mix of fixed and random-effects models based on heterogeneity. Results: A total of 21 studies met the inclusion criteria, encompassing 50,771 patients, with 21.75% undergoing robotic surgery and 78.25% laparoscopic surgery. Robotic surgery was associated with longer operative times (SMD = −1.27, *p* < 0.00001) but shorter hospital stays (MD = 0.42, *p* = 0.003) compared to laparoscopic surgery. Conversion rates were significantly higher in laparoscopic procedures (OR = 2.02, *p* < 0.00001). No significant differences were found in anastomotic leak rates. A higher number of lymph nodes was harvested by robotic approach (MD = −0.65, *p* = 0.04). Publication bias was addressed through funnel plot analysis and Egger’s test, indicating the presence of asymmetry (*p* = 0.006). Conclusions: The choice of surgical method should be individualized, considering factors such as surgeon expertise, medical facilities, and patient-specific considerations. Future research should aim to elucidate long-term outcomes to further guide the clinical decision-making.

## 1. Introduction

Colon cancer is a well-known pathology in the medical field, being one of the most prevalent malignancies and a leading cause of cancer-related mortality globally. Surgical intervention remains a cornerstone of colon cancer treatment, with minimally invasive techniques such as laparoscopic and robotic surgery becoming increasingly adopted due to the reduced postoperative pain, shorter hospital stays, and faster recovery. Continuous research and advancements in the surgical and oncological treatments are necessary to improve patient outcomes [1]. Minimally invasive surgery, including laparoscopic and robotic approaches, has played a significant role in the treatment of colon cancer, offering benefits such as reduced postoperative pain, shorter hospital stays, and fast recovery. While laparoscopic surgery has been widely used, robotic surgery has emerged as a promising alternative, claiming to enhance the accuracy of minimally invasive procedures with advanced maneuverability and other patient benefits, such as reduced complications [2,3,4].

Initial reports of laparoscopic colon resection appeared in early 1990s, and over three decades, the use of laparoscopy has increased to 40–50% of all colorectal resections for both benign and malignant conditions [5,6,7,8,9,10]. Robotic surgery received Food and Drug Administration (FDA) approval in July 2020 for various specialties, including general surgery. The first series of robotic colorectal surgery was documented in 2002 focusing on benign conditions, and it was followed by numerous studies comparing the laparoscopic and robotic approaches [11,12,13].

This study sought to answer the following question: What are the comparative effects of laparoscopic and robotic surgery on the outcomes of colon cancer treatment in terms of operative time, hospital stay, conversion rates, anastomotic leak rated, and oncological outcomes? To address this research question, we aimed to determine the outcomes by evaluating the objectives for each surgical approach and evaluate which method was more efficient.

In this study, we aimed to compare the operative outcomes of laparoscopic and robotic surgery for colon cancer, with a focus on operative time, hospital stay, conversion rates, anastomotic leak rates, and the total number of lymph nodes harvested. By evaluating and analyzing the latest studies published between 2020–2024, we aimed to provide insights into the benefits and drawbacks of each surgical approach, aiding surgeons and patients in making informed decisions regarding the most suitable surgical technique for colon cancer treatment.

## 2. Materials and Methods

A systematic review was conducted in accordance with the PRISMA (Preferred Reporting Items for Systematic Reviews and Meta-Analysis) guidelines [14]. The protocol was registered in PROSPERO database CRD42024513326, ensuring a structured and transparent review process. The study was designed to provide a comprehensive comparison of laparoscopic versus robotic surgery for colon cancer.

Inclusion criteria:

1. Study types: peer-reviewed randomized controlled trials and cohort studies;
2. Population: adult patients (aged 18 and older) diagnosed with colon cancer at any stage;
3. Interventions: studies comparing laparoscopic and robotic surgical techniques used specifically for colon cancer resections;
4. Outcomes: Studies must report at least one of the following outcomes: operative time, hospital stay, conversion rates, anastomotic leak rates, or harvested lymph nodes.

Exclusion criteria:

1. Non-comparative studies;
2. Cadaveric or animal studies;
3. Irrelevant conditions (other types of cancer or non-oncological surgeries);
4. Language restrictions;
5. Incomplete data (missing outcome data relevant to the primary endpoints of this review).

### 2.1. Search Strategy

The literature search was performed in January 2024, using four databases: Web of Science, SCOPUS, Science Direct, and PubMed. The search was made using the MeSH-term for greater precision [15]. The following terms were used: colonic neoplasm, colorectal neoplasms, colorectal tumor, colorectal tumors, minimal invasive surgical procedures, laparoscopic surgery, minimally invasive surgery, robotics, robotic surgery. The search included Boolean operators (AND, OR), using round and square brackets for the grouping of the search terms. The timeframe was filtered for articles published from January 2020 until the present (January 2024) to provide a contemporary analysis, considering the newest research and developments in the surgical field for colon cancer. Only the publication type “articles” was selected using the website filters, excluding any other type of publication (review article, proceeding papers, editorial material, early access, correction, letter, book chapters, etc.). The detailed search strategy can be found in Supplementary File S1.

### 2.2. Study Selection

The records were introduced on the Rayyan platform (Qatar Computing Research Institute) [16] for duplicate removal and a blind screening process by the two authors (N.R., C.A.). First evaluation of the records included a blind selection based on the title, keywords, and abstract. Any disagreement of the records screened was solved by discussion and by consulting the third reviewer (M.A.). The second screening included in-depth record evaluation. Any concerns of difference of opinion were solved by a group debate including the third reviewer.

The studies were included for assessment if they evaluated robotic and laparoscopic surgical approaches for colon cancer, including any stage 0/I/II/III/IV, and any location (caecum, ascending, transverse, descending, and sigmoid). If the paper reviewed in the same group colon and rectal cancer, it was excluded. If an article presented colon and rectal cancer, it was included only if the two groups were analyzed individually and data related to colon cancer could be extracted.

The studies were omitted following specific exclusion criteria:

1. Wrong publication type (review, meta-analysis);
2. Focusing on other diseases (rectal cancer, hepatic pathology, urologic-gynecologic pathology, gastric cancer, NOSES—natural orifice specimen extraction site, endometriosis, etc.);
3. Restricted access;
4. Animal or cadaveric study;
5. Foreign language;
6. No relevant data;
7. Missing data.

### 2.3. Data Extraction

Data extraction was performed by the researchers for the following study details: author names, publication year, research design, country where the study was conducted, and the timeframe for each study. Primary outcomes were operative time, length of hospital stay, conversion rate, anastomotic leak, and number of harvested lymph nodes. The secondary outcomes included overall complications, Clavien-Dindo classification I–IV, specimen size, distance from tumor to distal margin and proximal margin, margin rate positivity, 30-day mortality and 30-day readmission, and overall survival. Demographic data included age, number of male cases, BMI, ASA score, UICC (Union for International Cancer Control) stage, tumor location, and type of surgical procedure. Discrepancies in data extraction were resolved through discussion.

### 2.4. Assessment

Each of the studies included was independently assessed for the risk of bias and relevance by three authors (N.R., C.A., M.A.) using the Joanna Briggs Institute (JBI) Critical Appraisal Checklist [17]. The checklist consists in 11 questions that evaluate different study areas that might identify possible bias risk. Discrepancies among reviewers were solved by discussion and through agreement. The bias risk in individual studies was categorized based on specific thresholds: low risk of bias if there were 70% or more answers with “yes”, moderate risk for those with 50–69%, and high risk for studies with less than 50% affirmative responses [17].

### 2.5. Statistical Analysis

The statistical analysis was made in RevMan 5.4 provided by the Cochrane Collaboration [18]. For continuous variables, we calculated the mean difference (MD) or standardized mean difference (SMD) with 95% confidence intervals (CI), based on the scales used for measurements across the studies. For dichotomous variables, we calculated odds ratios (OR) or 95%CI to estimate effect size. Both fixed-effects and random-effects models were employed, depending on the detected heterogeneity among studies results. The heterogeneity across studies was calculated using I square statistics, chi-square tests, and Z tests for the overall effect. Tests were also performed to determine the presence of heterogeneity. As the *Cochrane Handbook for Systematic Reviews and Interventions* describes, the I square test was interpreted as follows: 0–40% might not be important, 30–60% may represent moderate heterogeneity, 50–90% may represent substantial heterogeneity, and 75–100% considerable heterogeneity [19].

Continuous variables that were initially reported as medians and ranges have been transformed into means and standard deviations, following the methodology proposed by Hozo et al. [20] and transformation methods by Wan et al. [21]. This conversion facilitates the application of parametric statistical analysis, which requires data to be presented as mean and standard deviation.

A fixed-effects model was used in studies with heterogeneity under 50%, while a random-effects model was used for studies with high heterogeneity.

Regarding the *p*-value, this was considered statistically significant if *p* was under 0.05. To mitigate the risk of publication bias, funnel plots were employed.

For the publication bias assessment, we used funnel plots for asymmetry and applied Egger’s regression rest.

All statistical analyses were conducted using RevMan 5.4 [18] and JASP Team (2024, version 0.18.3) software for additional analyses such as the Egger’s regression rest and Bayesian analysis.

## 3. Results

### 3.1. Study Selection

**Figure 1:**
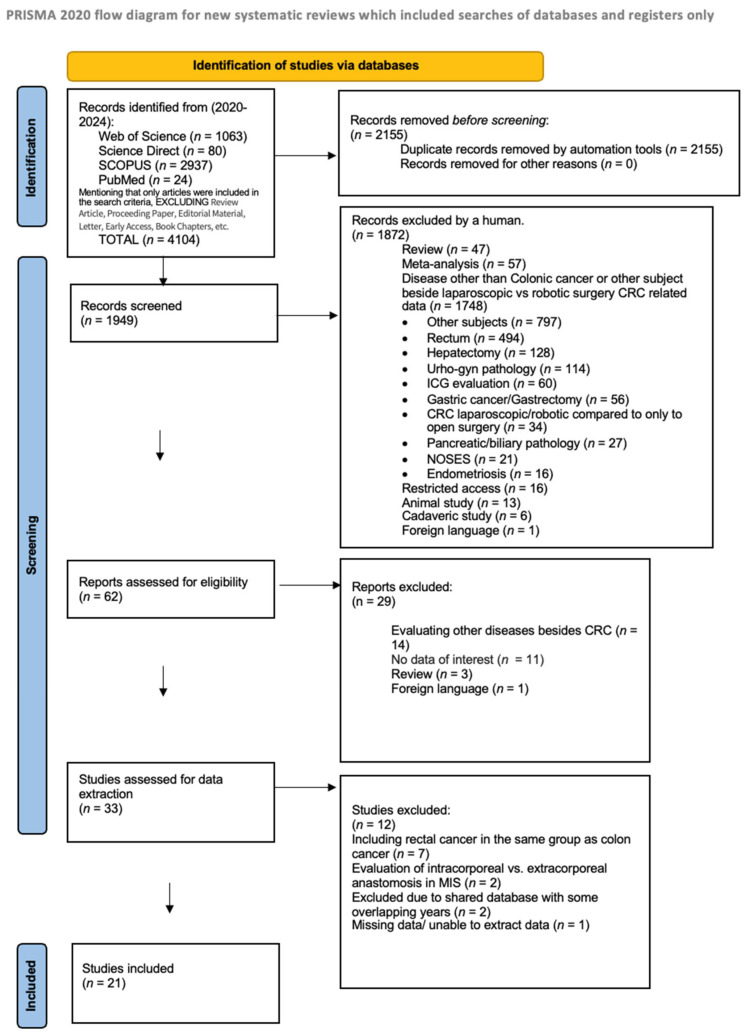
PRISMA flow diagram of studies selection.

The process of selected studies is synthetized in Figure 1, accordingly to PRISMA guidelines. At the beginning, after the systematic literature search, 4104 records were retrieved. After duplicate removal, 1949 studies were screened for title, keywords, and abstract. After the first screening, 61 studies were assessed for eligibility for the second screening process that meant complete text analysis. After that, the articles were assessed for data extraction. Following this, 21 articles were selected for inclusion in the quantitative analysis [22,23,24,25,26,27,28,29,30,31,32,33,34,35,36,37,38,39,40,41,42]. The identification of studies via databases and the inclusion and exclusion of the studies is presented in Figure 1.

### 3.2. Risk of Bias

Six studies were assessed as having moderate risk of bias, while the others were classified as having a low risk of bias according to the JBI Critical Appraisal Checklist (Table 1). The checklist can be found in Supplementary File S2.

### 3.3. Studies Characteristics

The characteristics of each study are shown in Table 2. A total of 50,771 patients were included from all studies; 11,059 of them were treated by robot-assisted surgery, and 39,712 were treated by the laparoscopic approach. Of the studies, six were from China, four from Italy, two from the United Kingdom, two from the United States, two from Korea, one from Slovenia, one from Denmark, one from Netherland, one from Spain, and one from Turkey.

### 3.4. Meta-Analysis

In the meta-analysis, we included 21 studies [22,23,24,25,26,27,28,29,30,31,32,33,34,35,36,37,38,39,40,41,42] out of 33 from the systematic literature search (see Figure 1). Of the total of 50.771 cases, 11.059 (21.75%) of them were treated by robot-assisted surgery, and 39.712 (78.25%) by the laparoscopic approach. For the meta-analysis, article 18 [38] had three subgroups, the first for right colectomy, the second for left colectomy, and the third for sigmoid resection, while article 19 [39] had two subgroups, the first for right colectomy, and the second for left colectomy, due to data distribution in the original research.

Analysis of the data related to patient demographics is presented in Table 3.

The statistical analysis is shown in Supplementary File S3.

**Figure 2:**
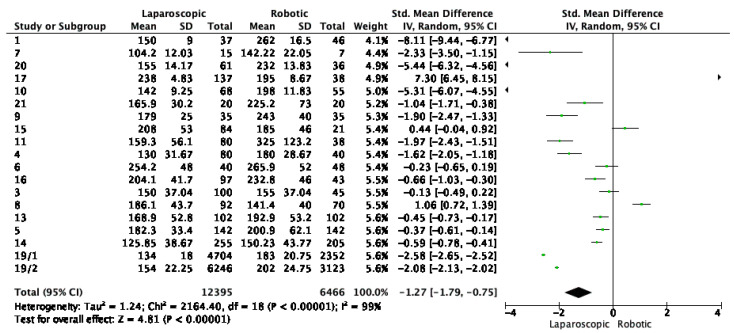
Forest plot for surgery time. Green dots represent point estimates of the mean difference between laparoscopic and robotic groups.

Primary outcomes:

For surgery duration, 18 studies were analyzed. The standardized mean difference (SMD) was −1.27 [−1.79, −0.75], indicating that laparoscopic surgery took significantly less time than robotic surgery. This difference was statistically significant, with a *p* < 0.00001 (Figure 2).

**Figure 3:**
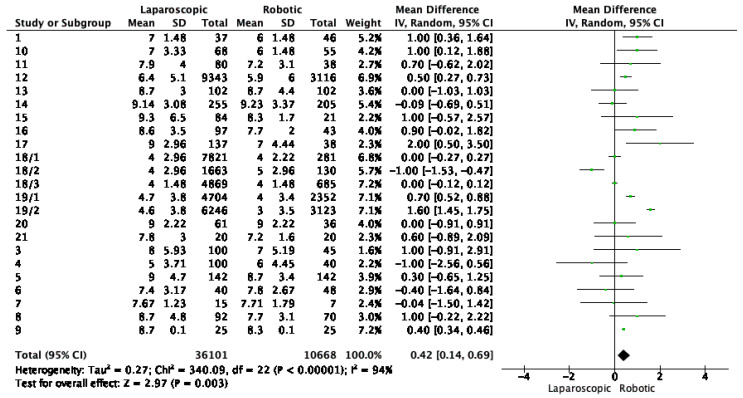
Forest plot for hospital stay. Green dots represent point estimates of the mean difference between laparoscopic and robotic groups.

Length of hospital stay (days) was reported in 20 studies, shown in Figure 3. The pooled data indicated a total mean difference of 0.42, meaning shorter hospitalization for robotic surgery, with a *p* value of 0.003 (Figure 3).

**Figure 4:**
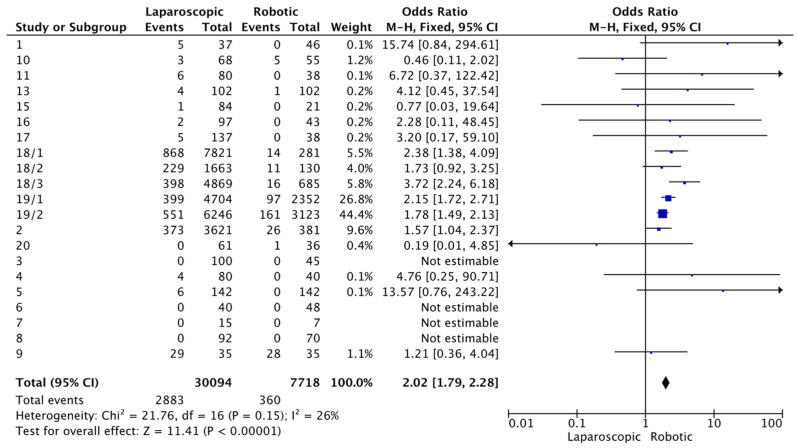
Forest plot for surgery conversion. Blue squares represent point estimate of odds ratio.

The analysis for conversion rates (Figure 4) when comparing laparoscopic to robotic surgery showed a total odds ratio of 2.02 (95%CI, [1.79, 2.28], which suggests the likelihood of surgery conversion was significantly higher for laparoscopic methods. The heterogeneity was low (I^2^ = 26%) and the overall effect was highly significant (Z = 11.41, *p* < 0.00001).

**Figure 5:**
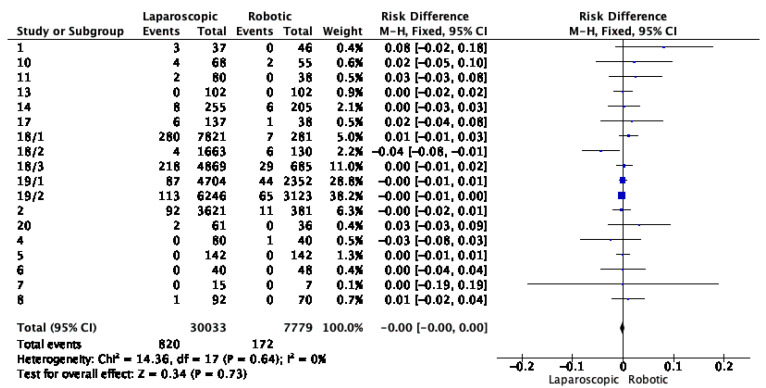
Risk difference for anastomotic leak between laparoscopic and robotic surgery. Blue squares represent point estimates of Risk Difference.

The pooled results for anastomotic leak between laparoscopic and robotic surgery showed no significant difference between the two methods, with a risk difference of −0.00 (95% CI [−0.00, 0.00]). The heterogeneity was non-existent, meaning no variation between studies, with an overall effect of −0.34 and a *p* value of 0.73, suggesting no statistically significant difference between the two surgical techniques. The results are shown in Figure 5.

**Figure 6:**
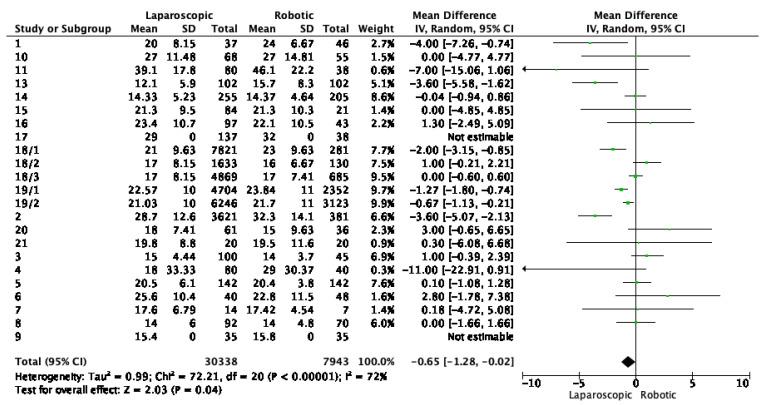
Lymphatic nodes harvested by laparoscopic and robotic-assisted approach. Green dots represent point estimates of the mean difference between laparoscopic and robotic groups.

Analyzing the mean number of harvested lymph nodes, the total mean difference was −0.65, indicating that on average, laparoscopic surgery resulted in 0.65 fewer lymph nodes harvested compared to robotic surgery. The heterogeneity was high (72%), suggesting substantial variation in outcomes across studies, a significant overall effect with a Z score of −2.03 and a *p*-value of 0.04. This indicates that robotic surgery was associated with a higher number of harvested lymphatic nodes. Results are shown in Figure 6.

Secondary outcomes:

Table 4 summarizes secondary outcomes from the meta-analysis, showing that none of the reported outcomes (specimen size, positive resection margins, distance from tumor to distal or proximal margin, complications, and major complications, 30-day mortality) showed a statistically significant difference between the two surgical techniques, as indicated by the *p*-values that were above the conventional threshold for significance of 0.05.

#### Publication Bias

**Figure 7:**
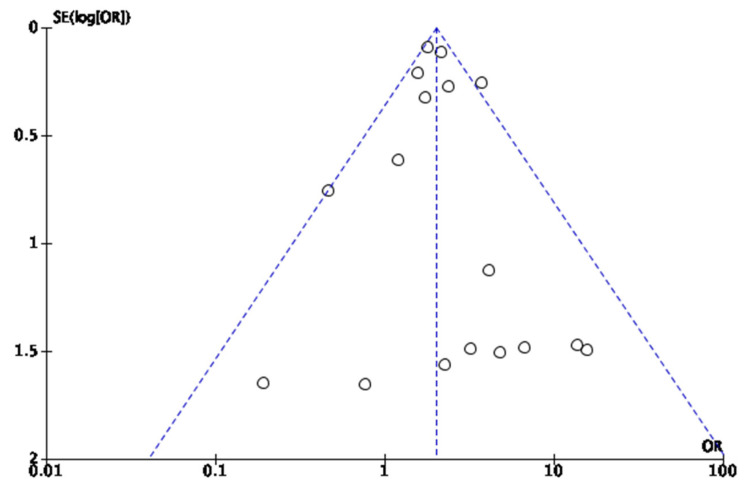
Funnel plot for conversion rates, used to ases publication bias.

We used a funnel plot of surgery conversion to estimate the presence of publication bias. The funnel plot displays a degree of asymmetry, with more studies being on the right side of the mean effect size line, suggesting potential publication bias (Figure 7).

Therefore, due to the asymmetry of the funnel plot, further analyses were conducted. For assessment of publication bias, JASP software [43] was used. Egger’s test was applied, showing a *p*-value of 0.006, which was below the 0.05 threshold, indicating significant funnel plot asymmetry, as shown in Table 5. The results for the precision-effect test—precision effect estimate are shown in Table 6. The results suggest that after adjusting for publication bias, there was no statistically significant effect detected by the PET-PEESE analysis.

A robust Bayesian analysis was conducted, and its results are shown in Table 7. The Bayesian approach revealed evidence of both heterogeneity and publication bias, with effect sizes uncertain and wide credibility intervals, suggesting there may have been an effect, but not one estimated with precision.

Publication bias represents a notable concern in the research field arising when studies with positive or statistically significant results are preferentially published over those with non-significant findings. This is also compounded by the small studies that report large effect sizes, which can distort the perceived efficacy of interventions. While comprehensive literature searches and statistical adjustments are employed to minimize this bias, it is challenging to fully correct due to various factors. Therefore, publication bias is a limitation that is acknowledged in interpreting the results of the meta-analysis.

## 4. Discussion

The findings of this systematic review and meta-analysis shed light on the comparative outcomes of robotic and laparoscopic surgery for colon cancer. Our analysis revealed that robotic surgery was associated with longer operative times compared to laparoscopic surgery, indicating a distinct operative time disadvantage. However, it is important to note that robotic surgery offered benefits in terms of reduced hospital stay and higher lymph node harvest. These findings suggest that while laparoscopic surgery may require more time in the operating room, it can contribute to shorter hospital stays and potentially improved oncological outcomes through a higher number of lymph nodes harvested.

The analysis of conversion rates indicated that laparoscopic surgery had a higher likelihood of conversion to open surgery compared to robotic methods, with an odds ratio of 2.02 (95%CT, [1.79, 2.28]). This suggests a statistically significant difference, supported by low heterogeneity (Z = 11.41, *p* < 0.00001). However, it is crucial to consider this information within a broader spectrum of surgical practice. Conversion from laparoscopic to an open approach should not be viewed as a shortfall of the laparoscopic method. Instead, it is often a reflection of prudent surgical judgment in which the primary concern is patient safety and optimal outcomes. Conversions are typically associated with intraoperative challenges such as unexpected anatomical complexities, technical difficulties or other patient factors that may not and cannot be fully appreciated preoperatively. By choosing to convert to an open procedure, when necessary, surgeons demonstrate adaptability and commitment to the best outcomes for the patient. Even though our study highlights a numerical difference in conversion rates, this should not be interpreted as a failure of the laparoscopic approach.

The operative time for laparoscopic surgery was significantly shorter than that for robotic surgery, due to subjective factors that might implicate the learning curve, the experience of the surgeon, but also the complexity of the case and because of the time needed for each instrument change [44,45]. This finding corroborates the work of previous meta-analyses that suggested efficiency in operative time as a key advantage of laparoscopic surgery [46]. It is pertinent to consider that extended operative times associated with robotic surgery may not reflect inefficiency but also encompass the learning curve for surgeons less experienced with robotic techniques. In this context, the robotic reduced-port approach has been recognized for its feasibility and safety across a spectrum of surgeons’ expertise, even among those with limited case volumes in single or reduced port surgeries [47].

In terms of anastomotic integrity, our meta-analysis focused on the critical comparison of anastomotic leak rates between the two surgical approaches. The results from pooled studies revealed a risk difference of −0.00 (95%CI [−0.00, 0.00]), indicating no significant discrepancy in the incidence of anastomotic leak. This finding underscores a consistent similarity in outcomes between the two minimal invasive surgical approaches, having a non-existent heterogeneity. While the incidence of anastomotic leaks did not differ between the two approaches, it remains mandatory for surgeons to continue to refine their techniques and decision-making to minimize this complication. Anastomotic healing is influenced by numerous factors, including tissue perfusion, surgical technique, and patient-related factors; the equivalent rates of anastomotic leaks suggest that both laparoscopic and robotic techniques are capable of achieving the standards of care necessary for optimal outcomes.

The lymphatic nodes harvest is a critical metric in oncologic surgery, serving as a marker for the thoroughness of the oncologic resection and impacting the staging accuracy. Our meta-analysis observed a total mean difference of −0.65, with laparoscopic surgery having on average 0.65 fewer lymph nodes retrieved, suggesting a slight advantage of robotic surgery. The heterogeneity of this result is high, indicating a considerable variability in the number of nodes harvested across different studies. This high heterogeneity could be due to multiple factors such as differences in surgical technique, the extent of the mesocolic excision, patient characteristics, tumor location, and even the interpretation of the examiner from the department of pathological anatomy. However, the difference of less than one lymph node on average may not translate into a clinically significant advantage.

Our review did not reveal any statistically significant oncological differences between laparoscopic and robotic surgery in terms of specimen size, positive resection margins, or distance from tumor to distal or proximal margin. These results suggest a parity between the two surgical approaches. The lack of statistically significant difference, with *p* values exceeding the conventional threshold of 0.05, indicates that both methods perform comparably across this metrics.

Similarly, the comparable rates of postoperative complications, major complications, and the 30-day mortality reflect the safety of both approaches. While the meta-analysis did not detect a difference in mortality between the two surgical techniques, it is important to mention and acknowledge that mortality is a multifaceted endpoint, that is influenced by many factors beyond surgical procedure itself.

The absence of significant differences further underscores the necessity for decision-making to be guided by surgeon expertise, resource availability, and patient factors. Future research with larger, more homogeneous study populations and long-term follow-up data is mandatory to validate this findings.

While this systematic review and meta-analysis was extensive, it has several limitations that must be considered when interpreting the findings:

1. Study design variability.

When both randomized control trials and cohort studies are included, heterogeneity is induced. Observational studies, in particular, may provide higher levels of bias compared to randomized trials.

2. Confounding factors

Unmeasured confounding factors such as surgeon expertise, patient selection, and hospital resources could influence the outcomes.

3. Geographical representation.

The studies included in the analysis do not cover all geographical regions.

4. Outcomes measured.

This review focused on short-term surgical outcomes.

5. Publication bias.

One limitation of our study is the presence of publication bias, as indicated by the funnel plot and Egger’s test. This suggests that there may be an overrepresentation of studies with positive results, which could potentially influence the overall findings and conclusions. Future research should aim to address this bias and include a more comprehensive range of studies to ensure a balanced and unbiased analysis.

The limitations of this review must be acknowledged. The inclusion of studies with various designs and quality, the conversion of medians to means for continuous variables, and the presence of publication bias may impact the validity of the conclusions. It emphasizes the necessity for more high-quality, randomized controlled trials with transparent reporting to better understand the comparative effectiveness of these surgical approaches.

Furthermore, it is important to acknowledge that the choice of surgical method should be individualized, considering factors such as surgeon expertise and patient-specific considerations. The decision-making process should weigh the advantages of reduced hospital stay and potentially improved oncological outcomes with the disadvantage of longer operative times.

The practical implications of this study extend beyond the data to inform clinical decision-making in the treatment of colon cancer. Our analysis suggests that robotic surgery, despite longer operative times, may confer the benefit of shorter hospital stays, which are critical considerations in surgical planning and resource allocation. The findings also highlight the importance of surgical expertise in both laparoscopic and robotic techniques. Training programs should continue to expand skill development in both modalities. Our study supports a tailored approach where surgical method selection is based on surgeon comfort and experience, as well as patient-specific factors. Patient selection for each surgical approach should be individualized, considering factors such as the patient’s overall health, tumor characteristics, and the potential for faster postoperative mobilization with robotic surgery, which could be particularly beneficial for patients with comorbidities that may be exacerbated by prolonged hospitalization.

Future research should be conducted, including more randomized trials and prospective cohort studies with standardized outcome measures to provide a clearer comparison between these surgical modalities. Additionally, further investigation into the long-term outcomes and cost-effectiveness of robotic versus laparoscopic surgery is warranted to inform practice guidelines. Further research could also benefit from including detailed subgroup analyses based on patient demographics, tumor characteristics, and surgeon experience. By expanding the research to include more diverse geographical areas, surgeons could gain insights into how regional differences in healthcare practices and infrastructure impact the surgical outcomes.

## 5. Conclusions

This systematic review and meta-analysis add to the existing literature by providing a contemporary analysis that includes recent advances in surgical techniques. While both robotic and laparoscopic surgeries are viable options for the treatment of colon cancer, the decision on which to choose should be guided by a multidisciplinary team to optimize patient outcomes. With the surgical field being in continuous development with technological advancements, ongoing evaluation and comparison of operative approaches remain essential.

The findings highlight that robotic surgery is associated with longer operative times but tends to result in shorter hospital stays.

The nuanced outcome of conversion rates further explains the complexity of surgical decision-making, reinforcing that conversion should not be deemed a failure of the laparoscopic technique but rather a strategic move towards ensuring patient safety and optimal surgical outcomes.

The equivalence observed in the outcomes such as specimen size, margin positivity, and the insignificant difference in the number of lymph nodes harvested, emphasizes that both laparoscopic and robotic surgeries meet the high standards required for oncological resection in colon cancer treatment.

The presence of publication bias, as indicated by the funnel plot asymmetry and Egger’s regression test, is a limitation of this study and the field at large, which can influence the generalizability of our findings.

This analysis calls for a more individualized approach to surgical method selection and underscores the imperative for ongoing, high-quality research to refine the comparative understanding of these surgical modalities. Future research should include more randomized trials and prospective cohort studies with standardized outcome measures and long follow-up periods to better compare long-term outcomes.

In summary, both surgical approaches are competent, showing no substantial differences in outcomes that would distinctly favor one technique over the other.

As we advance, it is crucial that we continue to critically assess and integrate new evidence to refine our surgical choices and enhance patient care.

## Figures and Tables

**Table 1:** Risk of bias.

Study	Q1	Q2	Q3	Q4	Q5	Q6	Q7	Q8	Q9	Q10	Q11	% YES	RISK	RISK
Jan Grosek et al., Slovenia, 2021 [22]	**Y**	**Y**	**Y**	**Y**	**Y**	**Y**	**Y**	**U**	**U**	**U**	**Y**	73%	☺	LOW
Niclas Dohrn et al. Denmark 2021 [23]	**Y**	**Y**	**Y**	**Y**	**Y**	**Y**	**Y**	**U**	**U**	**U**	**Y**	73%	☺	LOW
Yaqi Zhang et al., China 2022 [24]	**Y**	**Y**	**Y**	**Y**	**Y**	**Y**	**Y**	**Y**	**U**	**U**	**Y**	82%	☺	LOW
J. S. Khan et al., UK 2021 [25]	**Y**	**Y**	**Y**	**Y**	**Y**	**Y**	**Y**	**Y**	**U**	**U**	**Y**	82%	☺	LOW
Yue Tian et al., China 2023 [26]	**Y**	**U**	**U**	**Y**	**Y**	**Y**	**Y**	**Y**	**Y**	**U**	**Y**	73%	☺	LOW
Nadia Sorgato et al., Italy 2022 [27]	**Y**	**U**	**U**	**Y**	**Y**	**Y**	**Y**	**Y**	**Y**	**U**	**Y**	73%	☺	LOW
Alessandra Di Lascia et al., Italy 2020 [28]	**Y**	**Y**	**Y**	**U**	**U**	**Y**	**Y**	**U**	**U**	**U**	**Y**	55%	☺	MODERATE
Zhixiang Huang et al., China 2022 [29]	**Y**	**Y**	**Y**	**Y**	**Y**	**Y**	**Y**	**Y**	**U**	**U**	**Y**	82%	☺	LOW
Valentina Ferri et al., Spain 2020 [30]	**Y**	**U**	**U**	**Y**	**Y**	**Y**	**Y**	**Y**	**Y**	**U**	**Y**	73%	☺	LOW
Fulvio Tagliabue et al., Italy 2020 [31]	**Y**	**U**	**U**	**Y**	**Y**	**Y**	**Y**	**Y**	**U**	**U**	**Y**	64%	☺	MODERATE
V. Ozben et al., Turkey 2020 [32]	**Y**	**U**	**U**	**Y**	**Y**	**Y**	**Y**	**Y**	**Y**	**U**	**Y**	73%	☺	LOW
Filipe Pacheco et al., USA 2023 [33]	**Y**	**U**	**U**	**Y**	**Y**	**Y**	**Y**	**Y**	**U**	**U**	**Y**	64%	☺	MODERATE
Huichao Zeng et al., China 2023 [34]	**Y**	**Y**	**Y**	**Y**	**Y**	**Y**	**Y**	**Y**	**Y**	**Y**	**Y**	100%	☺	LOW
Maolin Xu et al., China 2020 [35]	**Y**	**Y**	**Y**	**Y**	**Y**	**Y**	**Y**	**Y**	**U**	**U**	**Y**	82%	☺	LOW
Tung-Cheng Chang et al., China 2021 [36]	**Y**	**Y**	**Y**	**Y**	**Y**	**Y**	**Y**	**U**	**U**	**U**	**Y**	73%	☺	LOW
Ho Segun Kim et al., Korea 2021 [37]	**Y**	**Y**	**Y**	**Y**	**Y**	**Y**	**Y**	**U**	**U**	**U**	**Y**	73%	☺	LOW
V. Maertens et al., UK 2022 [38]	**Y**	**Y**	**Y**	**U**	**Y**	**Y**	**Y**	**Y**	**U**	**U**	**Y**	73%	☺	LOW
Marlou F. M. Sterk et al., Netherland 2023 [39]	**Y**	**Y**	**Y**	**U**	**Y**	**Y**	**Y**	**U**	**U**	**U**	**Y**	64%	☺	MODERATE
Emile Farah et al., USA 2023 [40]	**Y**	**Y**	**Y**	**Y**	**Y**	**Y**	**Y**	**Y**	**U**	**U**	**Y**	82%	☺	LOW
Sung Uk Bae et al., Korea 2022 [41]	**Y**	**U**	**U**	**Y**	**Y**	**Y**	**Y**	**Y**	**U**	**U**	Y	64%	☺	MODERATE
Graziano Ceccarelli et al., Italy 2020 [42]	**Y**	**Y**	**Y**	**Y**	**Y**	**Y**	**Y**	**U**	**U**	**U**	**Y**	55%	☺	MODERATE

Green color for yes answers. Q1–Q11 refer to the JBI questions numerated from 1 to 11. Y means yes, U means uncertain.

**Table 2:** Study characteristics.

Author, Country, Year of Publication, Ref. Nr.	Nr	Study Type, Period of Study	Cases Number Lap/Rob	Propensity Score Matching	Sex Male Lap/Rob	Age Lap/Rob	BMI Lap/Rob
Jan Grosek et al., Slovenia 2021 [22]	1	Retrospective single center 2019–2020	37 LAP	-	23/26	67.5 ± 10.1/66.8 ± 11	27.2 (25.1–29.4)/27.5 (25.7–31.3)
			46 ROB				
Niclas Dohrn et al., Denmark 2021 [23]	2	Retrospective National Cancer Database 2015–2018	3621 LAP	-	1621/185	73 ± 8.89/73 ± 8.89	25.7 (22.9–29.04)/25.6 (23.2–28.8)
			381 ROB				
Yaqi Zhang et al., China 2022 [24]	3	Retrospective	100 LAP	1:3	41/19	-	-
		single center 2016–2018	45 ROB				
J. S. Khan et al., UK 2021 [25]	4	Prospective single center 2007–2017, 2014–2017	80 LAP	2:1			
			40 ROB		37/19	71 ± 33.3/69 ± 34.07	28 (19–47)/26 (20–37)
Yue Tian et al., China 2023 [26]	5	Retrospective multicenter 2016–2021	142 LAP	1:1	79/74	63.4 ± 11.3/63.2 ± 2.4	22.5 (3.24)/22.5 (3.2)
			142 ROB				
Nadia Sorgato et al., Italy 2022 [27]	6	Prospective multicenter 2018–2019	40 LAP	-	28/27	68 ± 10/71 ± 12.2	26.6 (17.9–36.3)/25.6 (17.5–47.3)
			48 ROB				
Alessandra Di Lascia et al., Italy 2020 [28]	7	Retrospective single center 2014–2017	15 LAP 7 ROB	-	8/4	75 ± 3/75.7 ± 2.56	25 (19–41)/26 (21–38)
Zhixiang Huang et al., China 2022 [29]	8	Retrospective single center 2012–2017	92 LAP 70 ROB	-	-	-	-
Valentina Ferri et al., Spain 2020 [30]	9	Prospective single center 2013–2017, 2014–2018	35 LAP 35 ROB	1:1	20/23	68.2 ± 8.67/69.6 ± 7	25 (20–34)/23 (19–31)
Fulvio Tagliabue et al., Italy 2020 [31]	10	Retrospective single center 2014–2019	68 LAP 55 ROB	-	40/32	-	24.81 (23.10–28.45)/24.31 (22.11–27.56)
V. Ozben et al., Turkey 2020 [32]	11	Retrospective multicenter 2011–2018	80 LAP 38 ROB	-	47/27	64.1 ± 15.5/62.3 ± 15.7	26.7 (7.7)/25.3 (6.1)
Filipe Pacheco et al., USA 2023 [33]	12	Retrospective National Cancer Database 2010–2018	9343 LAP 3116ROB	3:1	3957/1314	-	-
Huichao Zeng et al., China 2023 [34]	13	Retrospective multicenter 2014–2022	102 LAP 102 ROB	1:1	66/71	59 ± 12.5/61 ± 16.25	23.5 (4.86)/23.7 (4.46)
Maolin Xu et al., China 2020 [35]	14	Retrospective single center 2012–2018	255 LAP 205 ROB	-	170/123	60.26 ± 11.04/60.36 ± 11.33	24.78 (4.27)/24.8 (4.51)
Tung-Cheng Chang et al., China 2021 [36]	15	Retrospective multicenter 2013–2019	84 LAP 21 ROB	1:4	43/9	65.6 ± 13.6/62.1 ± 11.9	24.6 (4.19)/24.7 (5.27)
Ho Segun Kim et al., Korea 2021 [37]	16	Retrospective single center 2019–2022	97 LAP 43 ROB	-	63/12	70.6 ± 7.7/58.8 ± 7.7	24.3 (10.4)/23.4 (4.05)
V. Maertens et al., UK 2022 [38]	17	Retrospective single center 2005–2021	137 LAP 38 ROB	-	82/20	71 ± 9.17/65 ± 8.17	27 (18–40)/26.5 (20–33)
Marlou F. M. Sterk et al., Netherland 2023 [39]	18/1; 18/2; 18/3	Retrospective Dutch colorectal audit 2018–2020	14353 LAP 1096 ROB	-	Gr1:3557/136 Gr2:913/70 Gr3: 2886/405	Gr1: 73 ± 9.63/73 ± 9.63 Gr2:70 ± 10.37/71 ± 11.11 Gr3:69 ± 12.59/69 ± 11.85	Gr1:26.0 (23.4–29.1)/25.7 (23.4–28.9) Gr2: 26.2 (23.5–29.4)/26.7 (24.4–29.6) Gr3: 26.1 (23.6–29.1)/26.0 (23.7–28.7)
Emile Farah et al., USA 2023 [40]	19/1; 19/2	Retrospective ACS-NSQIP Database 2015–2020	10950 LAP 5475 ROB	2:1	Gr1:2187/1106 Gr2: 3266/1633	-	-
Sung Uk Bae et al., Korea, 2022 [41]	20	Retrospective single center 2014–2016	61 LAP 36 ROB	-	38/17	67 ± 10.37/62 ± 10.37	24.0 (21.0–27.0)/24.6 (21.0–27.0)
Graziano Ceccarelli et al., Italy, 2020 [42]	1	Retrospective single center 2014–2019	20 LAP 20 ROB	1:1	13/14	74.6 ( ± 13.8)/70.6 ( ± 9.9)	24.1 (22.14–26.06)/23 (21.38–24.62)

Values are expressed as mean-SD for age and median [IQR] for BMI. Lap—laparoscopic surgery, Rob—robotic-assisted surgery. Nr 1–22 refers to the numeration of each article in the meta-analysis.

**Table 3:** Patient demographics.

Outcome	Nr. of Studies	Lap	Rob	OR/MD (95%CI Interval)	I^2^ (%)	*p*-Value
Age mean(SD)	18	69.065 ± 10.577	67.96 ± 12.697	0.98 [0.01–1.95]	80%	0.05
Sex male	20	19,085 (48.16%)	5366 (48.81%)	0.96 [0.85, 1.08]	70%	0.48
ASA score > 3	18	12,863 (42.64%)	4033 (51.75%)	1.04 [0.98, 1.10]	33%	0.18
UICC stage III–IV	17	4737 (33.11%)	1443 (32.44%)	1.01 [0.94–1.09]	0%	0.80

OR—odds ratio, MD—mean difference, CI—confidence interval, I^2^—heterogeneity, Lap—laparoscopic surgery, Rob—robotic surgery, UICC Stage—Union for International Cancer Control Staging.

**Table 4:** Secondary outcomes meta-analysis results for laparoscopic vs. robotic surgery.

Laparoscopic vs. Robotic Surgery	Number of Studies	*p*-Value	I^2^	OR/MD (95%CI)	Chi^2^	Z
Specimen size	4	0.43	69%	−1.64 [−5.69, 2.42]	12.72	0.79
Positive resection margins	12	0.81	0%	−0.00 [−0.02, 0.01]	2.14	0.24
Distance from tumor to distal margin	4	0.79	0%	0.15 [−0.96, 1.26]	2.04	0.27
Distance from tumor to proximal margin	6	0.30	92%	1.52 [−1.35, 4.38]	66.61	1.04
Complications	18	0.44	0%	1.03 [0.95, 1.12]	17.01	0.78
Major complications Clavien-Dindo III–IV	17	0.98	0%	1.00 [0.89, 1.13]	7.18	0.03
30-day mortality	14	0.21	31%	0.00 [−0.00, 0.01]	20.38	1.26

**Table 5:** Egger’s test.

Regression Test for Funnel Plot Asymmetry (“Egger’s Test”)		
	Z	*p*
sei	2.733	0.006

**Table 6:** PET-PEESE analysis.

Mean Estimates (μ)							
						95% Confidence Interval	
	Estimate	Standard Error	t	df	*p*	Lower	Upper
PET	−0.076	0.061	−1.242	19	0.229	−0.196	0.044
PEESE	0.038	0.069	0.555	19	0.585	−0.097	0.173

**Table 7:** Robust Bayesian meta-analysis.

Summary				
	Models	P(M)	P(M|Data)	Inclusion BF
Effect	18/36	0.500	0.533	1.140
Heterogeneity	18/36	0.500	1.000	1.503 × 10^+125^
Publication bias	32/36	0.500	0.977	41.566

## Data Availability

Data are contained within the article and the Supplementary Materials.

## Supplementary Materials

The following supporting information can be downloaded at: https://www.mdpi.com/article/10.3390/cancers16081552/s1, Supplementary File S1: Search Strategy. Supplementary File S2: JBI Critical Appraisal Checklist. Supplementary File S3: Statistical Analysis. References [22,23,24,25,26,27,28,29,30,31,32,33,34,35,36,37,38,39,40,41,42] are cited in the Supplementary Materials.
