# Supplementary material for: Systematic Review and Meta-Analysis of Laparoscopic versus Robotic-Assisted Surgery for Colon Cancer: Efficacy, Safety, and Outcomes—A Focus on Studies from 2020–2024
Source: Cancers (Basel). 2024 Apr 18;16(8):1552. doi: 10.3390/cancers16081552 (PMC11048614; doi:10.3390/cancers16081552)
Supplement: Supplementary file 1 [file cancers-16-01552-s001.zip › S1.pdf]

Search Strategy
12.01.2024 ORA 22.00

WEB OF SCIENCE:

1. **((ALL=(Colonic Neoplasms) OR ALL=(Colorectal neoplasms) OR ALL=(Colorectal tumor) OR ALL=(Colorectal tumour) OR ALL=(Colonic tumour) OR ALL=(Colonic tumor)) AND (ALL=(Minimally Invasive Surgical Procedures) OR ALL=(Laparoscopic surgery) OR ALL=(Minimally invasive surgery)) AND (ALL=(robotics) OR ALL=(robotic surgery)))**

WOS:
TOTAL – 441
ARTICLE – 335

- Article335
- Review Article91
- Proceeding Paper19
- Editorial Material9
- Early Access8
- Correction2
- Letter2
- Book Chapters1

2020-2023: TOTAL - 229

- Article160
- Review Article52
- Early Access8
- Editorial Material4
- Proceeding Paper3
- Correction2

2. ((ALL=(COLONIC CANCER)) AND ALL=(ROBOTIC SURGERY)) AND ALL=(LAPAROSCOPIC SURGERY)

TOTAL- 117
ARTICLE - 92

2020-2023 TOTAL - 40
Article28
Review Article10
Early Access1
Editorial Material1

3. ((ALL=(COLORECTAL CANCER)) AND ALL=(ROBOTIC SURGERY)) AND ALL=(LAPAROSCOPIC SURGERY)
   TOTAL – 1241

ARTICLE- 914

2020-2024 – TOTAL 572

- Article410
- Review Article139
- Early Access33
- Letter9
- Editorial Material8
- Proceeding Paper7
- Meeting Abstract4
- Book Chapters2
- Correction2

4. ((ALL=(COLORECTAL CANCER)) AND (ALL=(ROBOTIC SURGERY) OR ALL=(Robotics)) AND (ALL=(LAPAROSCOPIC SURGERY) OR ALL=(Minimally Invasive Surgical Procedures) OR ALL=(Minimally invasive surgery)))

TOTAL- 644
ARTICLE – 465

2020-2024 TOTAL 644

- Article465
- Review Article153
- Early Access35
- Letter10
- Editorial Material8
- Proceeding Paper7
- Correction4
- Meeting Abstract4
- Book Chapters2

SCOPUS:

1. ((ALL ("Colonic Neoplasms") OR ALL ("Colorectal neoplasms") OR ALL ("Colorectal tumor") OR ALL ("Colorectal tumour") OR ALL ("Colonic tumour") OR ALL ("Colonic tumor")) AND (ALL ("Minimally Invasive Surgical Procedures") OR ALL ("Laparoscopic surgery") OR ALL ("Minimally invasive surgery")) AND (ALL ("robotics") OR ALL ("robotic surgery")))

TOTAL- 1253
ARTICLE- 843

2020-2024
TOTAL -628
ARTICLE -437

2. ALL ("COLONIC CANCER") AND ALL ("LAPAROSCOPIC SURGERY") AND ALL ("ROBOTIC SURGERY")

TOTAL 228
ARTICOLE 148

2020-2024
TOTAL – 118
ARTICOLE- 70

3. ((ALL ("COLORECTAL CANCER")) AND ALL ("ROBOTIC SURGERY")) AND ALL ("LAPAROSCOPIC SURGERY")

TOTAL – 2180
ARTICLE-1383

2020-2024
TOTAL- 1108
ARTICLE- 746

4. ALL("COLORECTAL CANCER") AND (ALL("ROBOTIC SURGERY") OR ALL("Robotics")) AND (ALL("LAPAROSCOPIC SURGERY") OR ALL("Minimally Invasive Surgical Procedures") OR ALL("Minimally invasive surgery"))

TOTAL -4951
ARTICLE- 3113

2020-2024
TOTAL- 2504
ARTICLE-1684

1. ((Colonic Neoplasms) OR (Colorectal neoplasms) OR (Colorectal tumor) OR (Colorectal tumour) OR (Colonic tumour) OR Colonic tumor)) AND ((Minimally Invasive Surgical Procedures) OR (Laparoscopic surgery) OR (Minimally invasive surgery)) AND ((robotics) OR (robotic surgery))

TOTAL – 1670
ARTICLE - 49

2020-2024:
TOTAL-864

ARTICLE- 19

SCIENCE DIRECT

1. Colorectal cancer, laparoscopic surgery, robotic surgery

TOTAL- 302
ARTICLE- 175

2020-2024
TOTAL - 143
ARTICLE -80

PubMed

((Colonic cancer) OR (Colorectal cancer) OR (Colonic Neoplasms) OR (Colorectal neoplasms) OR (Colorectal tumor) OR (Colorectal tumour) OR (Colonic tumour) OR (Colonic tumor)) AND ((Minimally Invasive Surgical Procedures) OR (Laparoscopic surgery) OR (Minimally invasive surgery)) AND ((robotics) OR (robotic surgery))

Total- 1941
Article- 56

2020-2024

Total- 1034
Article- 24
