# Supplementary material for: Systematic Review and Meta-Analysis of Laparoscopic versus Robotic-Assisted Surgery for Colon Cancer: Efficacy, Safety, and Outcomes—A Focus on Studies from 2020–2024
Source: Cancers (Basel). 2024 Apr 18;16(8):1552. doi: 10.3390/cancers16081552 (PMC11048614; doi:10.3390/cancers16081552)
Supplement: Supplementary file 1 [file cancers-16-01552-s001.zip › S2.pdf]

Study	Q1	Q2	Q3	Q4	Q5	Q6	Q7	Q8	Q9	Q10	Q11	% YES	RISK	RISK
Jan Grosek, et al. Slovenia,2021[22]	Y	U	U	Y	Y	Y	Y	U	U	U	Y	55%		MODERATE
Niclas Dohrn et al. Denmark,2021 [23]	Y	U	U	Y	Y	Y	Y	N	N	N	Y	55%	5	MODERATE
Yaqi Zhang et al. China,2022 [24]	Y	U	Y	Y	Y	Y	Y	Y	U	U	Y	73%	3	LOW
J. S. Khan et al. UK,2021 [25]	Y	Y	Y	Y	Y	Y	Y	Y	U	U	Y	82%	2	LOW
Yue Tian et al. China, 2023 [26]	Y	U	U	Y	Y	Y	Y	Y	Y	U	Y	73%		LOW
Nadia Sorgato et al. Italy,2022 [27]	Y	U	U	Y	Y	Y	Y	Y	Y	U	Y	73%		LOW
Alessandra Di Lascia et al. Italy,2020 [28]	Y	Y	Y	U	U	Y	Y	U	U	U	Y	55%		MODERATE
Zhixiang Huang et al. China, 2022 [29]	Y	Y	Y	Y	Y	Y	Y	Y	U	U	Y	82%		LOW
Valentina Ferri et al. Spain, 2020 [30]	Y	U	U	Y	Y	Y	Y	Y	Y	U	Y	73%		LOW
Fulvio Tagliabue et al. Italy, 2020 [31]	Y	U	U	Y	Y	Y	Y	Y	U	U	Y	64%	4	MODERATE
V. Ozben et al. Turkey, 2020 [32]	Y	U	U	Y	Y	Y	Y	Y	Y	U	Y	73%		LOW
Filipe Pacheco et al. USA, 2023 [33]	Y	U	U	Y	Y	Y	Y	Y	U	U	Y	64%		MODERATE
Huichao Zeng et al. China, 2023 [34]	Y	Y	Y	Y	Y	Y	Y	Y	Y	Y	Y	100%		LOW
Maolin Xu et al. China, 2020 [35]	Y	Y	Y	Y	Y	Y	Y	Y	U	U	Y	82%		LOW
Tung-Cheng Chang et al. China,2021 [36]	Y	Y	Y	Y	Y	Y	Y	U	U	U	Y	73%		LOW
Ho Segun Kim et al. Korea, 2021 [37]	Y	Y	Y	Y	Y	Y	Y	U	U	U	Y	73%		LOW
V. Maertens et al.	Y	Y	Y	U	Y	Y	Y	Y	U	U	Y	73%		LOW

UK, 2022 [38]														
Marlou F. M. Sterk et al. Netherland, 2023 [39]	Y	Y	Y	U	Y	Y	Y	U	U	U	Y	64%		MODERATE
Emile Farah et al. USA, 2023 [40]	…..													
Sung Uk Bae et al. Korea, 2022 [41]	…..													
Graziano Ceccarelli et al., Italy, 2020 [42]	…..													

1. Were the two groups similar and recruited from the same population?
2. Were the exposures measured similarly to assign people to both exposed and unexposed groups?
3. Was the exposure measured in a valid and reliable way?
4. Were confounding factors identified?
5. Were strategies to deal with confounding factors stated?
6. Were the groups/participants free of the outcome at the start of the study (or at the moment of exposure)?
7. Were the outcomes measured in a valid and reliable way?
8. Was the follow up time reported and sufficient to be long enough for outcomes to occur?
9. Was follow up complete, and if not, were the reasons to loss to follow up described and explored?
10. Were strategies to address incomplete follow up utilized?

11. Was appropriate statistical analysis used?
