# Supplementary material for: Systematic Review and Meta-Analysis of Laparoscopic versus Robotic-Assisted Surgery for Colon Cancer: Efficacy, Safety, and Outcomes—A Focus on Studies from 2020–2024
Source: Cancers (Basel). 2024 Apr 18;16(8):1552. doi: 10.3390/cancers16081552 (PMC11048614; doi:10.3390/cancers16081552)
Supplement: Supplementary file 1 [file cancers-16-01552-s001.zip › S3.pdf]

Study or Subgroup	Laparoscopic Events	Laparoscopic Total	Robotic Events	Robotic Total	Weight	Risk Difference M-H, Fixed, 95% CI
10	0	68	0	55	0.5%	0.00 [-0.03, 0.03]
11	0	80	0	38	0.4%	0.00 [-0.04, 0.04]
12	477	9343	130	3116	35.0%	0.01 [0.00, 0.02]
13	0	102	0	102	0.8%	0.00 [-0.02, 0.02]
15	0	84	0	21	0.3%	0.00 [-0.06, 0.06]
19/1	45	4704	16	2352	23.5%	0.00 [-0.00, 0.01]
19/2	48	6246	29	3123	31.2%	-0.00 [-0.01, 0.00]
2	50	3621	8	381	5.2%	-0.01 [-0.02, 0.01]
20	0	61	9	36	0.3%	-0.25 [-0.39, -0.11]
3	1	100	0	45	0.5%	0.01 [-0.03, 0.05]
4	0	80	0	40	0.4%	0.00 [-0.04, 0.04]
5	0	142	0	142	1.1%	0.00 [-0.01, 0.01]
6	0	40	0	48	0.3%	0.00 [-0.04, 0.04]
7	0	15	0	7	0.1%	0.00 [-0.19, 0.19]
8	0	92	0	70	0.6%	0.00 [-0.02, 0.02]
**Total (95% CI)**		**24778**		**9576**	**100.0%**	**0.00 [-0.00, 0.01]**
Total events	621		192			

Heterogeneity: $Chi^2 = 20.38$, df = 14 (P = 0.12); $I^2 = 31\%$
Test for overall effect: Z = 1.26 (P = 0.21)

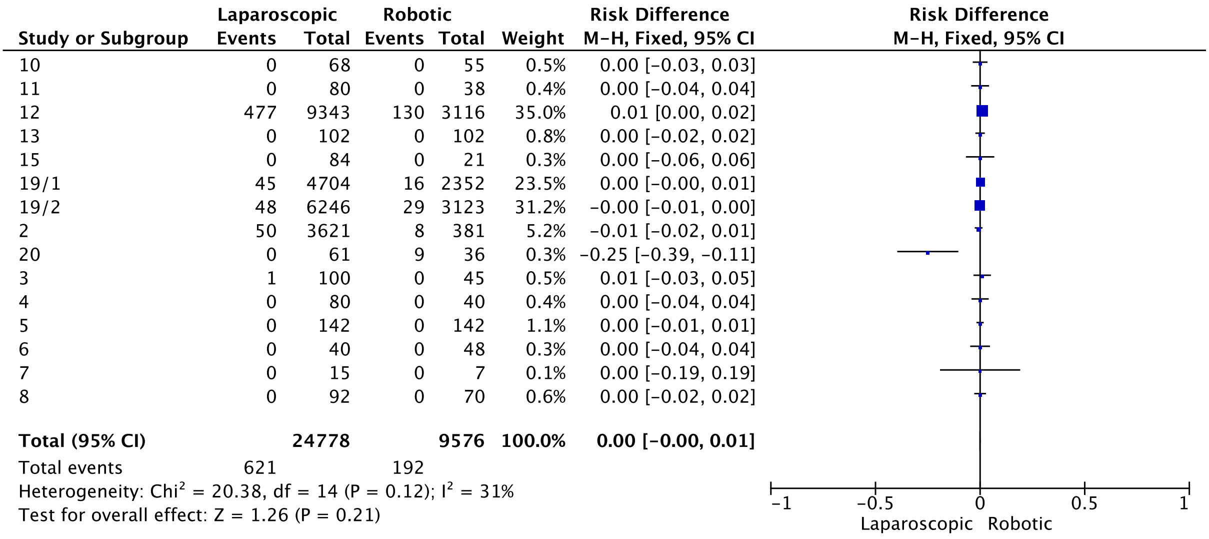

Study or Subgroup	Laparoscopic Mean	Laparoscopic SD	Laparoscopic Total	Robotic Mean	Robotic SD	Robotic Total	Weight	Mean Difference IV, Random, 95% CI
1	67.5	10.1	37	66.8	11	46	3.1%	0.70 [-3.85, 5.25]
10	72	11.5	68	72	10.39	55	3.8%	0.00 [-3.87, 3.87]
11	64.1	15.5	80	62.3	15.7	38	2.0%	1.80 [-4.24, 7.84]
12	84	5	9343	84	5	3116	9.7%	0.00 [-0.20, 0.20]
13	59	12.5	102	61	16.25	102	3.7%	-2.00 [-5.98, 1.98]
14	60.26	11.04	255	60.36	11.33	205	6.7%	-0.10 [-2.16, 1.96]
15	65.6	13.6	84	62.1	11.9	21	2.1%	3.50 [-2.36, 9.36]
16	70.6	7.7	97	58.8	7.7	43	5.4%	11.80 [9.04, 14.56]
17	71	9.7	137	65	8.17	38	4.9%	6.00 [2.94, 9.06]
18/1	73	9.63	3557	73	9.63	281	8.5%	0.00 [-1.17, 1.17]
18/2	70	10.37	913	71	11.11	130	6.8%	-1.00 [-3.02, 1.02]
18/3	69	12.59	2886	69	11.85	685	8.8%	0.00 [-1.00, 1.00]
2	73	8.89	3621	73	8.89	381	8.9%	0.00 [-0.94, 0.94]
20	67	10.37	61	62	10.37	36	3.4%	5.00 [0.73, 9.27]
21	74.6	13.8	20	70.6	9.9	20	1.4%	4.00 [-3.44, 11.44]
4	71	33.3	80	69	34.07	40	0.5%	2.00 [-10.83, 14.83]
5	63.4	11.3	142	63.2	2.4	142	7.1%	0.20 [-1.70, 2.10]
6	68	10	40	71	12.2	48	3.0%	-3.00 [-7.64, 1.64]
7	75	3	15	75.7	2.56	7	6.0%	-0.70 [-3.13, 1.73]
9	68.2	8.67	35	69.6	7	35	4.0%	-1.40 [-5.09, 2.29]
Total (95% CI)			21573			5469	100.0%	0.98 [0.01, 1.95]

Heterogeneity: $Tau^2 = 2.49$; $Chi^2 = 96.86$, df = 19 (P < 0.00001); $I^2 = 80\%$
Test for overall effect: Z = 1.99 (P = 0.05)

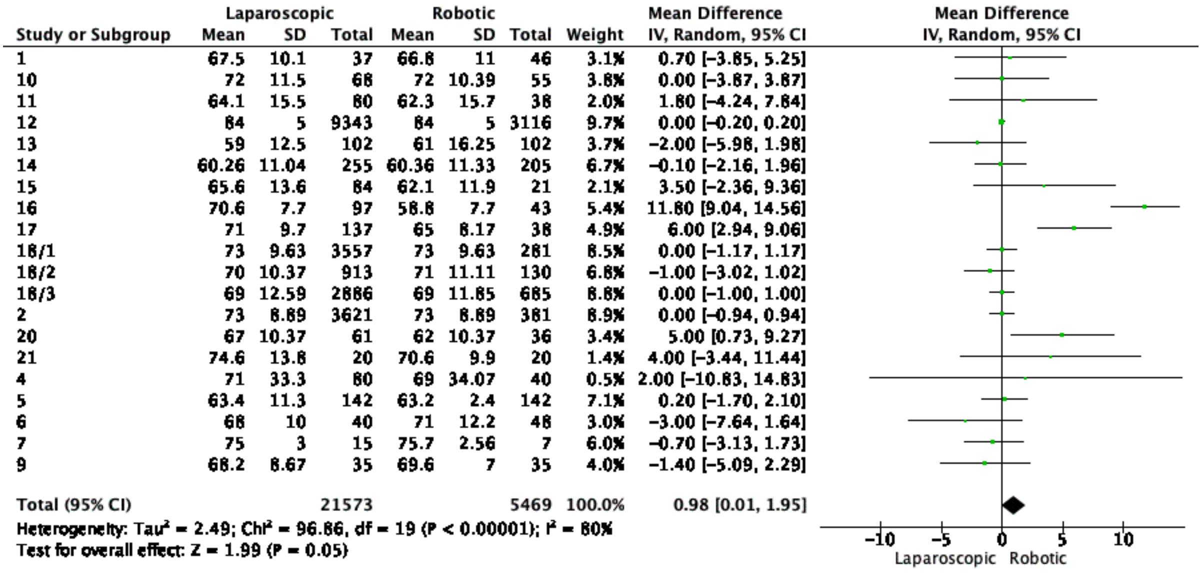

Study or Subgroup	Laparoscopic Mean	Laparoscopic SD	Laparoscopic Total	Robotic Mean	Robotic SD	Robotic Total	Weight	Mean Difference IV, Random, 95% CI
1	67.5	10.1	37	66.8	11	46	3.1%	0.70 [-3.85, 5.25]
10	72	11.5	68	72	10.39	55	3.8%	0.00 [-3.87, 3.87]
11	64.1	15.5	80	62.3	15.7	38	2.0%	1.80 [-4.24, 7.84]
12	84	5	9343	84	5	3116	9.7%	0.00 [-0.20, 0.20]
13	59	12.5	102	61	16.25	102	3.7%	-2.00 [-5.98, 1.98]
14	60.26	11.04	255	60.36	11.33	205	6.7%	-0.10 [-2.16, 1.96]
15	65.6	13.6	84	62.1	11.9	21	2.1%	3.50 [-2.36, 9.36]
16	70.6	7.7	97	58.8	7.7	43	5.4%	11.80 [9.04, 14.56]
17	71	9.7	137	65	8.17	38	4.9%	6.00 [2.94, 9.06]
18/1	73	9.63	3557	73	9.63	281	8.5%	0.00 [-1.17, 1.17]
18/2	70	10.37	913	71	11.11	130	6.8%	-1.00 [-3.02, 1.02]
18/3	69	12.59	2886	69	11.85	685	8.8%	0.00 [-1.00, 1.00]
2	73	8.89	3621	73	8.89	381	8.9%	0.00 [-0.94, 0.94]
20	67	10.37	61	62	10.37	36	3.4%	5.00 [0.73, 9.27]
21	74.6	13.8	20	70.6	9.9	20	1.4%	4.00 [-3.44, 11.44]
4	71	33.3	80	69	34.07	40	0.5%	2.00 [-10.83, 14.83]
5	63.4	11.3	142	63.2	2.4	142	7.1%	0.20 [-1.70, 2.10]
6	68	10	40	71	12.2	48	3.0%	-3.00 [-7.64, 1.64]
7	75	3	15	75.7	2.56	7	6.0%	-0.70 [-3.13, 1.73]
9	68.2	8.67	35	69.6	7	35	4.0%	-1.40 [-5.09, 2.29]
**Total (95% CI)**			**21573**			**5469**	**100.0%**	**0.98 [0.01, 1.95]**

Heterogeneity: $Tau^2 = 2.49$; $Chi^2 = 96.86$, df = 19 (P < 0.00001); $I^2 = 80\%$
Test for overall effect: Z = 1.99 (P = 0.05)

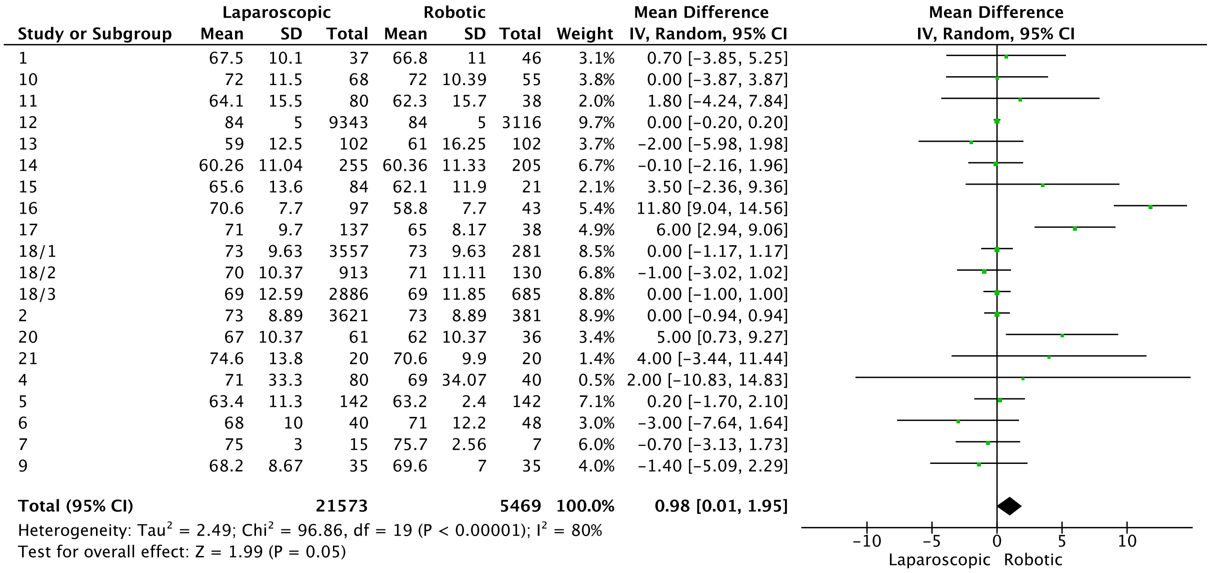

Study or Subgroup	Laparoscopic Events	Laparoscopic Total	Robotic Events	Robotic Total	Weight	Risk Difference M-H, Fixed, 95% CI
1	3	37	0	46	0.4%	0.08 [−0.02, 0.18]
10	4	68	2	55	0.6%	0.02 [−0.05, 0.10]
11	2	80	0	38	0.5%	0.03 [−0.03, 0.08]
13	0	102	0	102	0.9%	0.00 [−0.02, 0.02]
14	8	255	6	205	2.1%	0.00 [−0.03, 0.03]
17	6	137	1	38	0.5%	0.02 [−0.04, 0.08]
18/1	280	7821	7	281	5.0%	0.01 [−0.01, 0.03]
18/2	4	1663	6	130	2.2%	−0.04 [−0.08, −0.01]
18/3	218	4869	29	685	11.0%	0.00 [−0.01, 0.02]
19/1	87	4704	44	2352	28.8%	−0.00 [−0.01, 0.01]
19/2	113	6246	65	3123	38.2%	−0.00 [−0.01, 0.00]
2	92	3621	11	381	6.3%	−0.00 [−0.02, 0.01]
20	2	61	0	36	0.4%	0.03 [−0.03, 0.09]
4	0	80	1	40	0.5%	−0.03 [−0.08, 0.03]
5	0	142	0	142	1.3%	0.00 [−0.01, 0.01]
6	0	40	0	48	0.4%	0.00 [−0.04, 0.04]
7	0	15	0	7	0.1%	0.00 [−0.19, 0.19]
8	1	92	0	70	0.7%	0.01 [−0.02, 0.04]
**Total (95% CI)**		**30033**		**7779**	**100.0%**	**−0.00 [−0.00, 0.00]**
Total events	820		172			

Heterogeneity: $Chi^2 = 14.36$, $df = 17$ ($P = 0.64$); $I^2 = 0\%$
Test for overall effect: $Z = 0.34$ ($P = 0.73$)

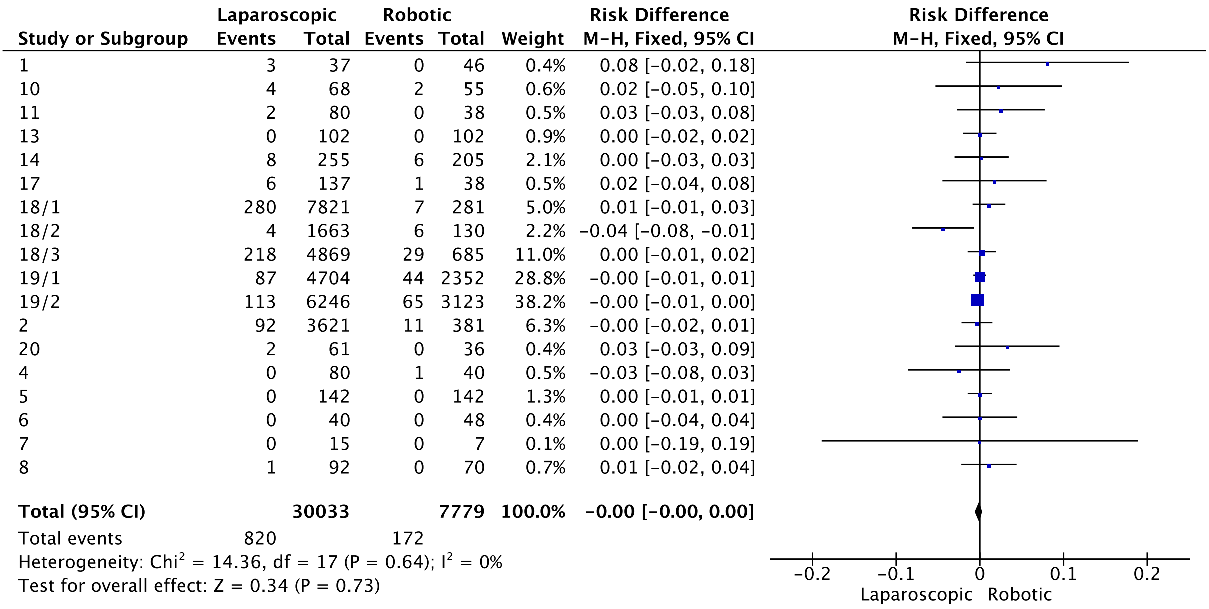

Study or Subgroup	Laparoscopic Events	Laparoscopic Total	Robotic Events	Robotic Total	Weight	Risk Difference M-H, Fixed, 95% CI
1	3	37	0	46	0.4%	0.08 [−0.02, 0.18]
10	4	68	2	55	0.6%	0.02 [−0.05, 0.10]
11	2	80	0	38	0.5%	0.03 [−0.03, 0.08]
13	0	102	0	102	0.9%	0.00 [−0.02, 0.02]
14	8	255	6	205	2.1%	0.00 [−0.03, 0.03]
17	6	137	1	38	0.5%	0.02 [−0.04, 0.08]
18/1	280	7821	7	281	5.0%	0.01 [−0.01, 0.03]
18/2	4	1663	6	130	2.2%	−0.04 [−0.08, −0.01]
18/3	218	4869	29	685	11.0%	0.00 [−0.01, 0.02]
19/1	87	4704	44	2352	28.8%	−0.00 [−0.01, 0.01]
19/2	113	6246	65	3123	38.2%	−0.00 [−0.01, 0.00]
2	92	3621	11	381	6.3%	−0.00 [−0.02, 0.01]
20	2	61	0	36	0.4%	0.03 [−0.03, 0.09]
4	0	80	1	40	0.5%	−0.03 [−0.08, 0.03]
5	0	142	0	142	1.3%	0.00 [−0.01, 0.01]
6	0	40	0	48	0.4%	0.00 [−0.04, 0.04]
7	0	15	0	7	0.1%	0.00 [−0.19, 0.19]
8	1	92	0	70	0.7%	0.01 [−0.02, 0.04]
Total (95% CI)		30033		7779	100.0%	−0.00 [−0.00, 0.00]
Total events	820		172			

Heterogeneity: $Chi^2 = 14.36$, $df = 17$ ($P = 0.64$); $I^2 = 0\%$
Test for overall effect: $Z = 0.34$ ($P = 0.73$)

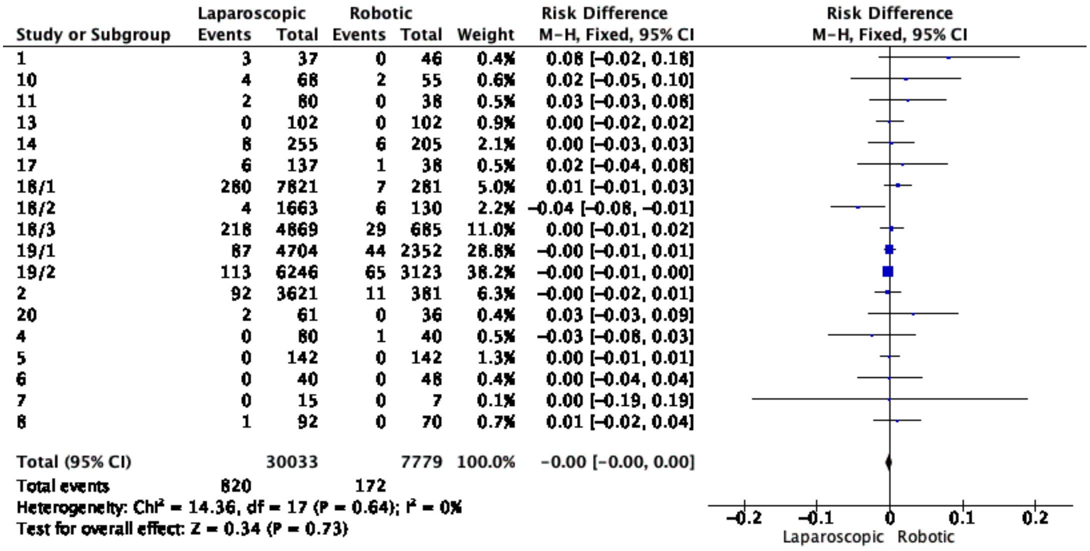

Study or Subgroup	Laparoscopic Events	Laparoscopic Total	Robotic Events	Robotic Total	Weight	Risk Difference M-H, Fixed, 95% CI
1	3	37	0	46	0.4%	0.08 [−0.02, 0.18]
10	4	68	2	55	0.6%	0.02 [−0.05, 0.10]
11	2	80	0	38	0.5%	0.03 [−0.03, 0.08]
13	0	102	0	102	0.9%	0.00 [−0.02, 0.02]
14	8	255	6	205	2.1%	0.00 [−0.03, 0.03]
17	6	137	1	38	0.5%	0.02 [−0.04, 0.08]
18/1	280	7821	7	281	5.0%	0.01 [−0.01, 0.03]
18/2	4	1663	6	130	2.2%	−0.04 [−0.08, −0.01]
18/3	218	4869	29	685	11.0%	0.00 [−0.01, 0.02]
19/1	87	4704	44	2352	28.8%	−0.00 [−0.01, 0.01]
19/2	113	6246	65	3123	38.2%	−0.00 [−0.01, 0.00]
2	92	3621	11	381	6.3%	−0.00 [−0.02, 0.01]
20	2	61	0	36	0.4%	0.03 [−0.03, 0.09]
4	0	80	1	40	0.5%	−0.03 [−0.08, 0.03]
5	0	142	0	142	1.3%	0.00 [−0.01, 0.01]
6	0	40	0	48	0.4%	0.00 [−0.04, 0.04]
7	0	15	0	7	0.1%	0.00 [−0.19, 0.19]
8	1	92	0	70	0.7%	0.01 [−0.02, 0.04]
**Total (95% CI)**		**30033**		**7779**	**100.0%**	**−0.00 [−0.00, 0.00]**
Total events	820		172			

Heterogeneity: $Chi^2 = 14.36$, $df = 17$ ($P = 0.64$); $I^2 = 0\%$
Test for overall effect: $Z = 0.34$ ($P = 0.73$)

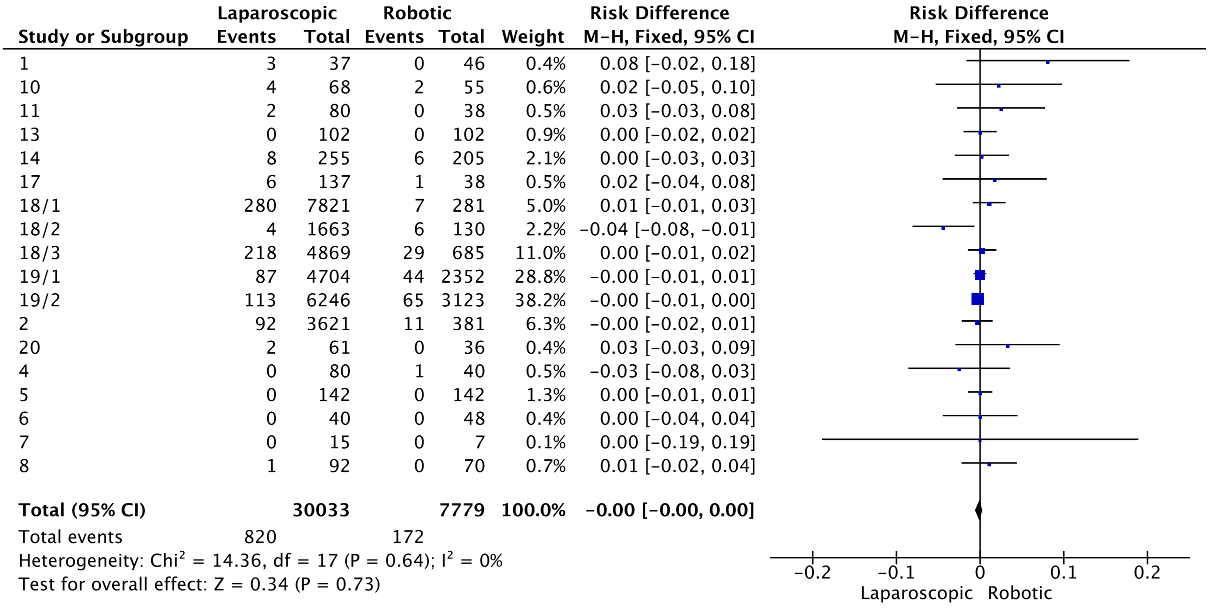

Study or Subgroup	Laparoscopic Events	Laparoscopic Total	Robotic Events	Robotic Total	Weight	Odds Ratio M-H, Fixed, 95% CI
1	17	37	20	46	0.4%	1.10 [0.46, 2.64]
10	21	68	14	55	0.5%	1.31 [0.59, 2.90]
11	25	80	9	38	0.4%	1.46 [0.60, 3.55]
13	0	102	2	102	0.1%	0.20 [0.01, 4.14]
14	24	255	26	205	1.1%	0.72 [0.40, 1.29]
15	8	84	1	21	0.1%	2.11 [0.25, 17.83]
16	36	97	2	43	0.1%	12.10 [2.76, 53.03]
17	33	137	6	38	0.3%	1.69 [0.65, 4.40]
18/1	2904	7821	92	281	4.9%	1.21 [0.94, 1.56]
18/2	514	1633	41	130	2.3%	1.00 [0.68, 1.46]
18/3	1285	4869	156	685	8.8%	1.22 [1.01, 1.47]
19/1	3106	4704	1555	2352	30.6%	1.00 [0.90, 1.11]
19/2	3867	6246	1933	3123	42.7%	1.00 [0.92, 1.09]
2	916	3621	97	381	5.7%	0.99 [0.78, 1.26]
20	4	61	3	36	0.2%	0.77 [0.16, 3.66]
21	6	20	7	20	0.2%	0.80 [0.21, 3.00]
3	2	100	2	45	0.1%	0.44 [0.06, 3.22]
4	24	80	7	40	0.3%	2.02 [0.78, 5.20]
6	13	40	11	48	0.3%	1.62 [0.63, 4.16]
8	50	92	46	70	1.0%	0.62 [0.33, 1.18]
9	8	35	3	35	0.1%	3.16 [0.76, 13.11]
**Total (95% CI)**		**30182**		**7794**	**100.0%**	**1.04 [0.98, 1.10]**
Total events	12863		4033			

Heterogeneity: $Chi^2 = 29.81$, df = 20 (P = 0.07); $I^2 = 33\%$
Test for overall effect: Z = 1.34 (P = 0.18)

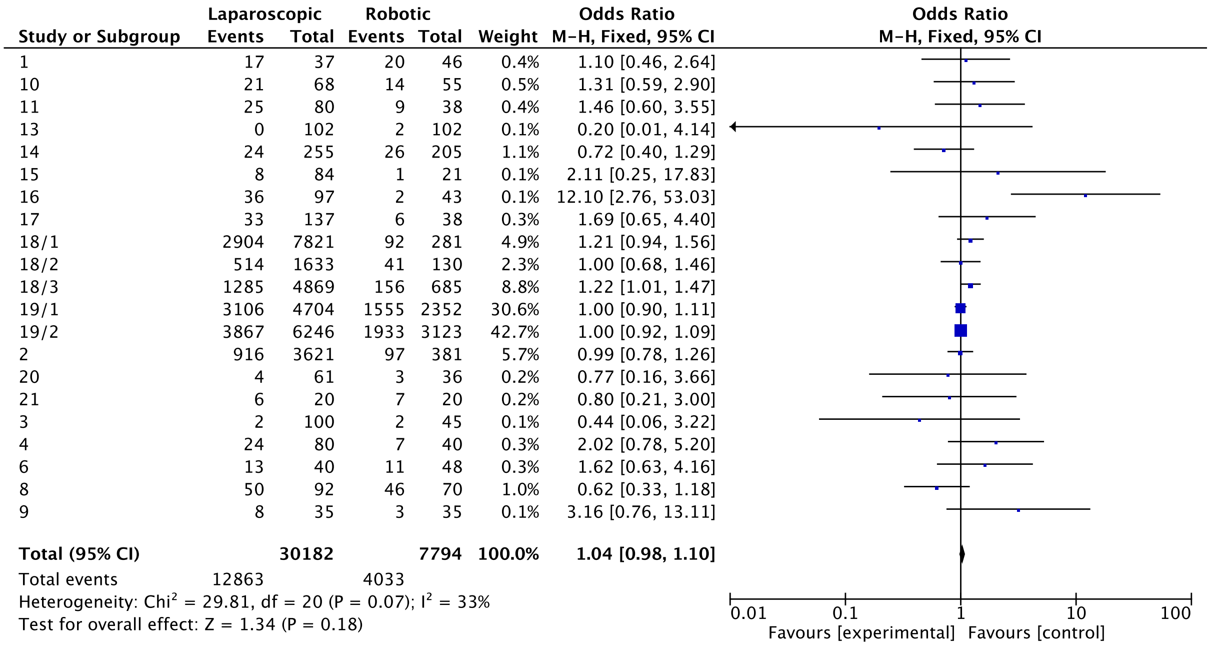

Study or Subgroup	Laparoscopic Events	Laparoscopic Total	Robotic Events	Robotic Total	Weight	Odds Ratio M-H, Fixed, 95% CI
1	10	37	10	46	0.5%	1.33 [0.49, 3.66]
10	23	68	17	55	1.0%	1.14 [0.53, 2.45]
11	23	80	13	38	1.1%	0.78 [0.34, 1.77]
13	18	102	19	102	1.3%	0.94 [0.46, 1.91]
14	59	255	54	205	3.9%	0.84 [0.55, 1.29]
15	6	84	1	21	0.1%	1.54 [0.18, 13.52]
16	24	97	7	43	0.6%	1.69 [0.67, 4.29]
17	41	137	10	38	0.9%	1.20 [0.53, 2.69]
19/1	779	4704	375	2352	35.0%	1.05 [0.91, 1.20]
19/2	870	6246	398	3123	38.3%	1.11 [0.98, 1.26]
2	760	3621	102	381	12.2%	0.73 [0.57, 0.92]
20	14	61	6	36	0.5%	1.49 [0.52, 4.30]
21	5	20	9	20	0.6%	0.41 [0.11, 1.56]
4	14	80	6	40	0.6%	1.20 [0.42, 3.41]
5	23	142	22	142	1.5%	1.05 [0.56, 1.99]
6	30	40	35	48	0.7%	1.11 [0.43, 2.90]
7	1	15	1	7	0.1%	0.43 [0.02, 8.04]
8	18	92	8	70	0.6%	1.89 [0.77, 4.63]
9	9	35	8	35	0.5%	1.17 [0.39, 3.49]
**Total (95% CI)**		**15916**		**6802**	**100.0%**	**1.03 [0.95, 1.12]**
Total events	2727		1101			

Heterogeneity: $Chi^2 = 17.01$, df = 18 (P = 0.52); $I^2 = 0\%$
Test for overall effect: Z = 0.78 (P = 0.44)

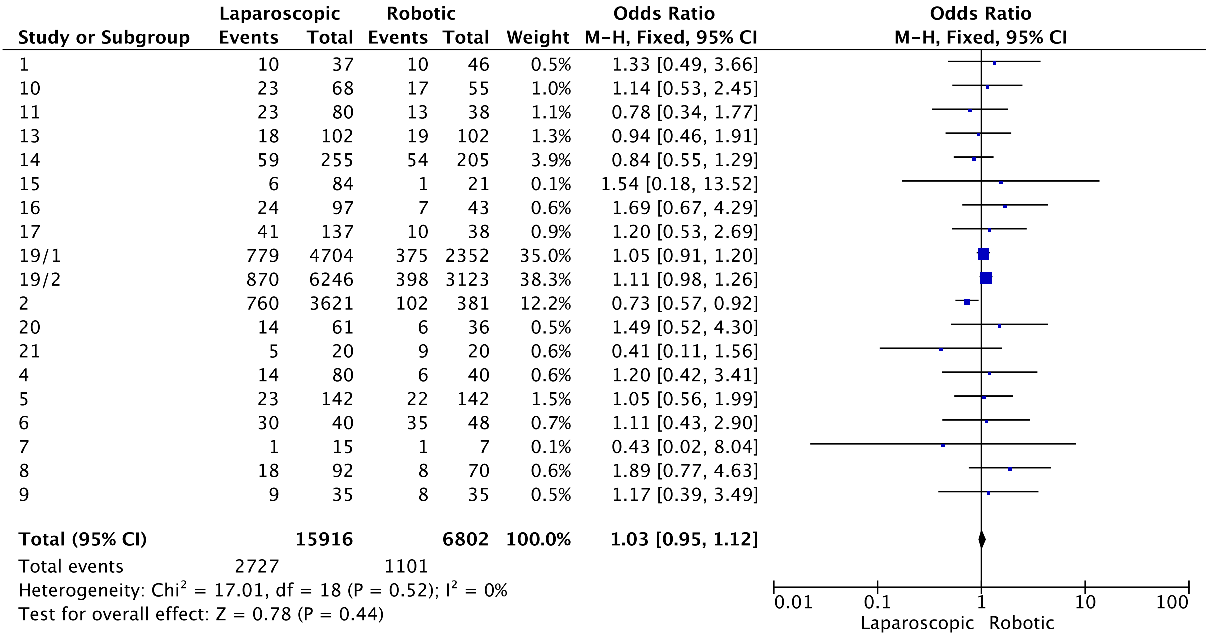

Study or Subgroup	Laparoscopic Events	Laparoscopic Total	Robotic Events	Robotic Total	Weight	Odds Ratio M-H, Fixed, 95% CI
6	0	40	0	48		Not estimable
7	0	15	0	7		Not estimable
8	0	92	0	70		Not estimable
3	0	100	0	45		Not estimable
1	5	37	0	46	0.1%	15.74 [0.84, 294.61]
5	6	142	0	142	0.1%	13.57 [0.76, 243.22]
11	6	80	0	38	0.1%	6.72 [0.37, 122.42]
4	4	80	0	40	0.1%	4.76 [0.25, 90.71]
16	2	97	0	43	0.2%	2.28 [0.11, 48.45]
17	5	137	0	38	0.2%	3.20 [0.17, 59.10]
15	1	84	0	21	0.2%	0.77 [0.03, 19.64]
13	4	102	1	102	0.2%	4.12 [0.45, 37.54]
20	0	61	1	36	0.4%	0.19 [0.01, 4.85]
9	29	35	28	35	1.1%	1.21 [0.36, 4.04]
10	3	68	5	55	1.2%	0.46 [0.11, 2.02]
18/2	229	1663	11	130	4.0%	1.73 [0.92, 3.25]
18/1	868	7821	14	281	5.5%	2.38 [1.38, 4.09]
18/3	398	4869	16	685	5.8%	3.72 [2.24, 6.18]
2	373	3621	26	381	9.6%	1.57 [1.04, 2.37]
19/1	399	4704	97	2352	26.8%	2.15 [1.72, 2.71]
19/2	551	6246	161	3123	44.4%	1.78 [1.49, 2.13]
**Total (95% CI)**		**30094**		**7718**	**100.0%**	**2.02 [1.79, 2.28]**
Total events	2883		360			

Heterogeneity: $Chi^2 = 21.76$, df = 16 (P = 0.15); $I^2 = 26\%$
Test for overall effect: Z = 11.41 (P < 0.00001)

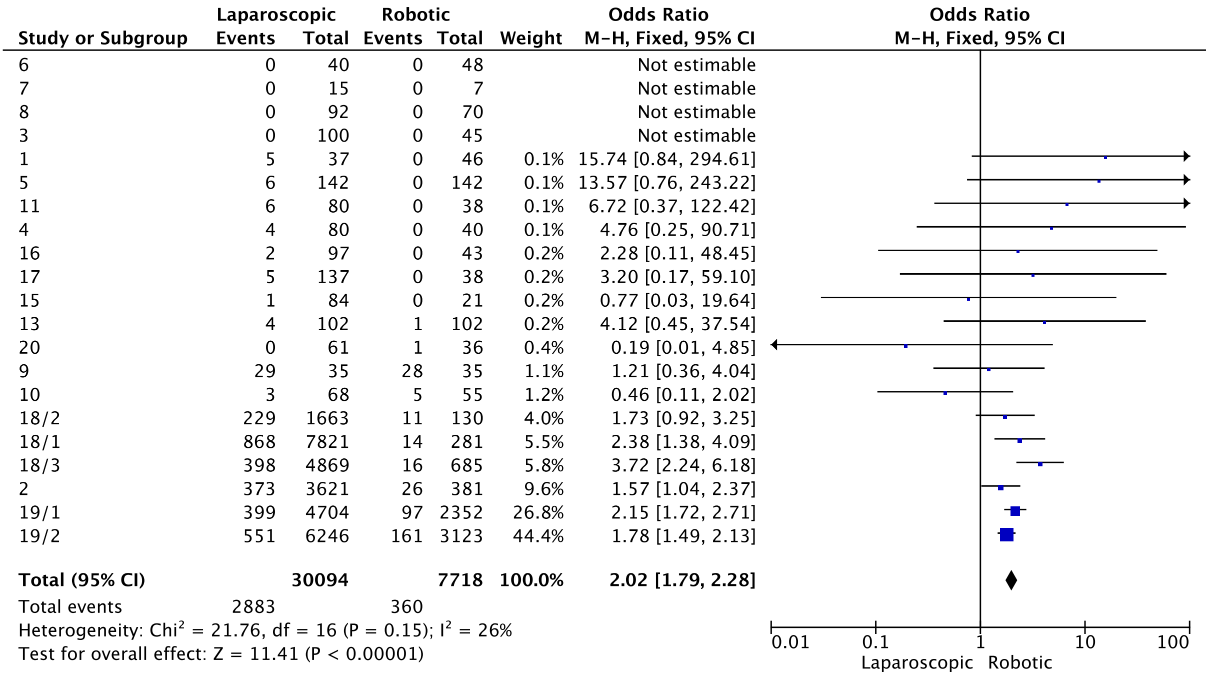

Study or Subgroup	Laparoscopic Mean	Laparoscopic SD	Laparoscopic Total	Robotic Mean	Robotic SD	Robotic Total	Weight	Mean Difference IV, Random, 95% CI
11	13.9	12.9	80	16.4	10.3	38	13.6%	-2.50 [-6.83, 1.83]
15	9.2	6.7	84	6.5	6.1	21	16.2%	2.70 [-0.28, 5.68]
16	11.3	7.3	97	1.8	6.2	43	17.3%	9.50 [7.15, 11.85]
20	3.7	1.93	61	4	3.26	36	19.0%	-0.30 [-1.47, 0.87]
4	5	0.5	80	5.5	2.96	40	19.2%	-0.50 [-1.42, 0.42]
6	15.1	8.4	40	15.6	9.7	48	14.6%	-0.50 [-4.28, 3.28]
**Total (95% CI)**			**442**			**226**	**100.0%**	**1.52 [-1.35, 4.38]**

Heterogeneity: $Tau^2 = 10.88$; $Chi^2 = 66.61$, df = 5 (P < 0.00001); $I^2 = 92\%$
Test for overall effect: Z = 1.04 (P = 0.30)

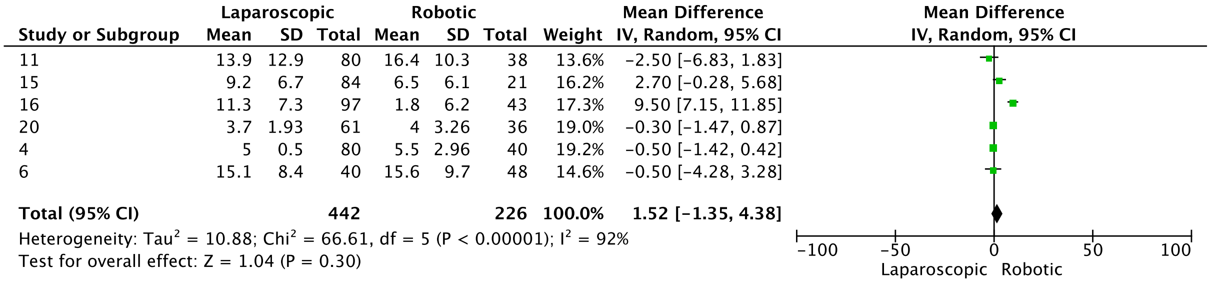

Study or Subgroup	Laparoscopic Mean	Laparoscopic SD	Laparoscopic Total	Robotic Mean	Robotic SD	Robotic Total	Weight	Mean Difference IV, Random, 95% CI
3	8	5.93	100	7	5.19	45	1.6%	1.00 [-0.91, 2.91]
15	9.3	6.5	84	8.3	1.7	21	2.2%	1.00 [-0.57, 2.57]
4	5	3.71	100	6	4.45	40	2.2%	-1.00 [-2.56, 0.56]
17	9	2.96	137	7	4.44	38	2.3%	2.00 [0.50, 3.50]
21	7.8	3	20	7.2	1.6	20	2.3%	0.60 [-0.89, 2.09]
7	7.67	1.23	15	7.71	1.79	7	2.4%	-0.04 [-1.50, 1.42]
11	7.9	4	80	7.2	3.1	38	2.7%	0.70 [-0.62, 2.02]
6	7.4	3.17	40	7.8	2.67	48	2.9%	-0.40 [-1.64, 0.84]
8	8.7	4.8	92	7.7	3.1	70	3.0%	1.00 [-0.22, 2.22]
13	8.7	3	102	8.7	4.4	102	3.6%	0.00 [-1.03, 1.03]
5	9	4.7	142	8.7	3.4	142	3.9%	0.30 [-0.65, 1.25]
16	8.6	3.5	97	7.7	2	43	4.0%	0.90 [-0.02, 1.82]
20	9	2.22	61	9	2.22	36	4.0%	0.00 [-0.91, 0.91]
10	7	3.33	68	6	1.48	55	4.2%	1.00 [0.12, 1.88]
1	7	1.48	37	6	1.48	46	5.2%	1.00 [0.36, 1.64]
14	9.14	3.08	255	9.23	3.37	205	5.4%	-0.09 [-0.69, 0.51]
18/2	4	2.96	1663	5	2.96	130	5.7%	-1.00 [-1.53, -0.47]
18/1	4	2.96	7821	4	2.22	281	6.8%	0.00 [-0.27, 0.27]
12	6.4	5.1	9343	5.9	6	3116	6.9%	0.50 [0.27, 0.73]
19/1	4.7	3.8	4704	4	3.4	2352	7.1%	0.70 [0.52, 0.88]
19/2	4.6	3.8	6246	3	3.5	3123	7.1%	1.60 [1.45, 1.75]
18/3	4	1.48	4869	4	1.48	685	7.2%	0.00 [-0.12, 0.12]
9	8.7	0.1	25	8.3	0.1	25	7.2%	0.40 [0.34, 0.46]
**Total (95% CI)**			**36101**			**10668**	**100.0%**	**0.42 [0.14, 0.69]**

Heterogeneity: $Tau^2 = 0.27$; $Chi^2 = 340.09$, df = 22 (P < 0.00001); $I^2 = 94\%$
Test for overall effect: Z = 2.97 (P = 0.003)

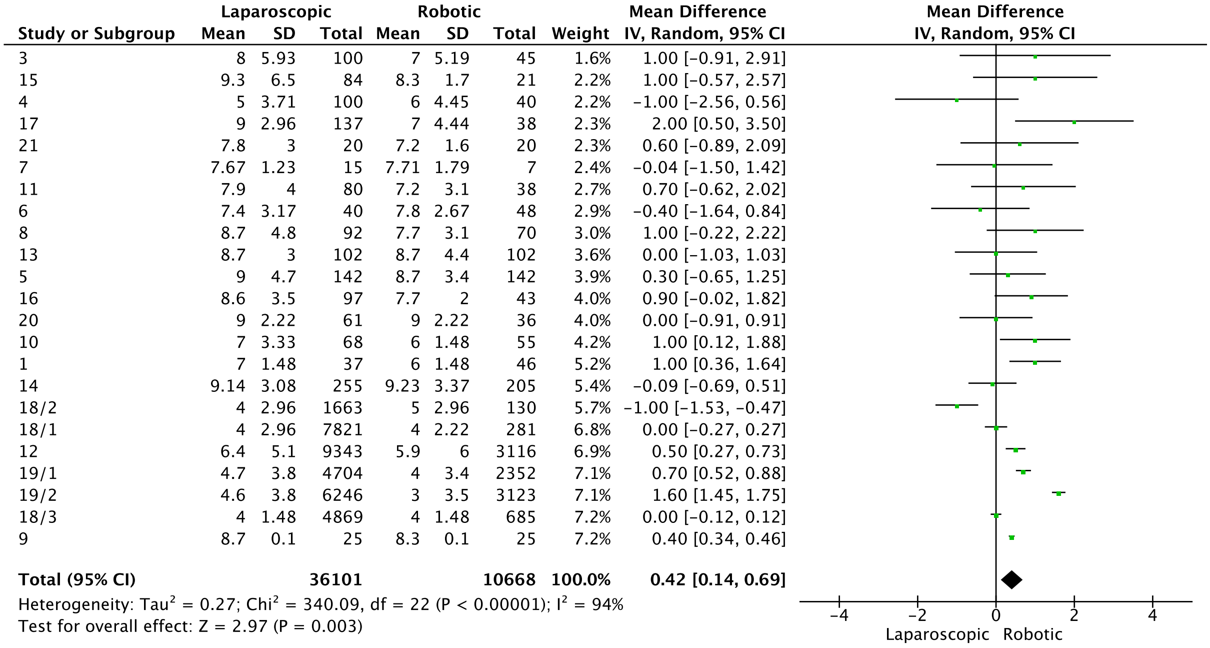

Study or Subgroup	Laparoscopic Mean	SD	Total	Robotic Mean	SD	Total	Weight	Mean Difference IV, Random, 95% CI
1	7	1.48	37	6	1.48	46	5.2%	1.00 [0.36, 1.64]
10	7	3.33	68	6	1.48	55	4.2%	1.00 [0.12, 1.88]
11	7.9	4	80	7.2	3.1	38	2.7%	0.70 [−0.62, 2.02]
12	6.4	5.1	9343	5.9	6	3116	6.9%	0.50 [0.27, 0.73]
13	8.7	3	102	8.7	4.4	102	3.6%	0.00 [−1.03, 1.03]
14	9.14	3.08	255	9.23	3.37	205	5.4%	−0.09 [−0.69, 0.51]
15	9.3	6.5	84	8.3	1.7	21	2.2%	1.00 [−0.57, 2.57]
16	8.6	3.5	97	7.7	2	43	4.0%	0.90 [−0.02, 1.82]
17	9	2.96	137	7	4.44	38	2.3%	2.00 [0.50, 3.50]
18/1	4	2.96	7821	4	2.22	281	6.8%	0.00 [−0.27, 0.27]
18/2	4	2.96	1663	5	2.96	130	5.7%	−1.00 [−1.53, −0.47]
18/3	4	1.48	4869	4	1.48	685	7.2%	0.00 [−0.12, 0.12]
19/1	4.7	3.8	4704	4	3.4	2352	7.1%	0.70 [0.52, 0.88]
19/2	4.6	3.8	6246	3	3.5	3123	7.1%	1.60 [1.45, 1.75]
20	9	2.22	61	9	2.22	36	4.0%	0.00 [−0.91, 0.91]
21	7.8	3	20	7.2	1.6	20	2.3%	0.60 [−0.89, 2.09]
3	8	5.93	100	7	5.19	45	1.6%	1.00 [−0.91, 2.91]
4	5	3.71	100	6	4.45	40	2.2%	−1.00 [−2.56, 0.56]
5	9	4.7	142	8.7	3.4	142	3.9%	0.30 [−0.65, 1.25]
6	7.4	3.17	40	7.8	2.67	48	2.9%	−0.40 [−1.64, 0.84]
7	7.67	1.23	15	7.71	1.79	7	2.4%	−0.04 [−1.50, 1.42]
8	8.7	4.8	92	7.7	3.1	70	3.0%	1.00 [−0.22, 2.22]
9	8.7	0.1	25	8.3	0.1	25	7.2%	0.40 [0.34, 0.46]
**Total (95% CI)**			**36101**			**10668**	**100.0%**	**0.42 [0.14, 0.69]**

Heterogeneity: Tau² = 0.27; Chi² = 340.09, df = 22 (P < 0.00001); I² = 94%
Test for overall effect: Z = 2.97 (P = 0.003)

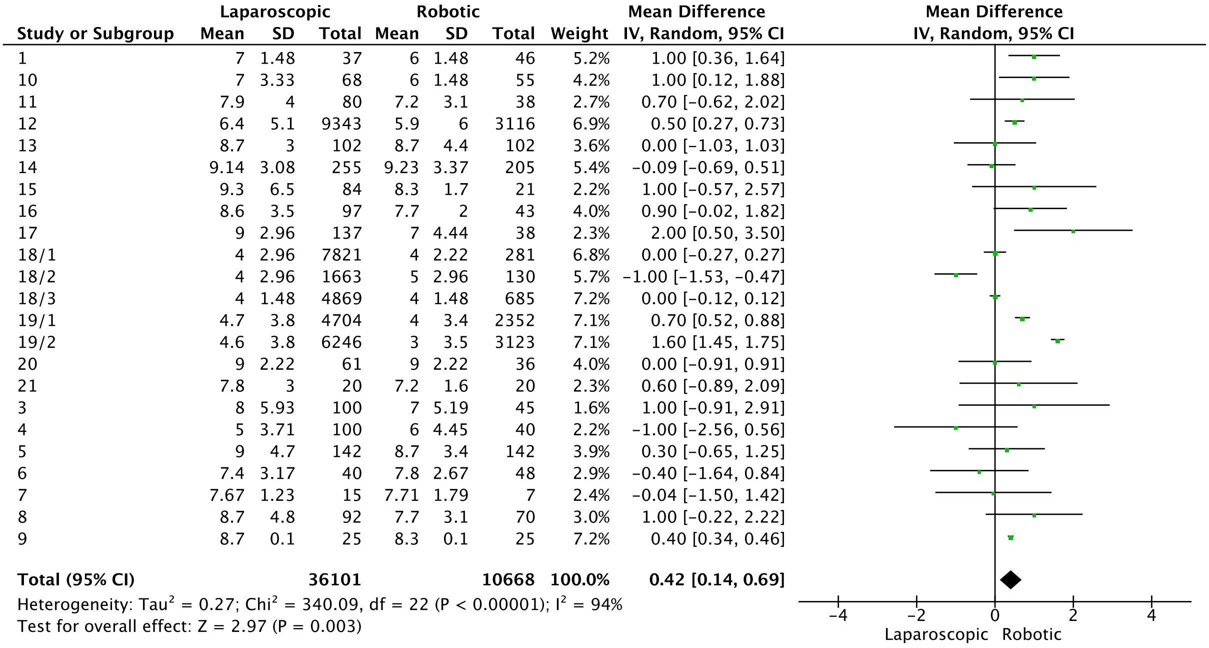

Study or Subgroup	Laparoscopic Mean	SD	Total	Robotic Mean	SD	Total	Weight	Mean Difference IV, Random, 95% CI
9	15.4	0	35	15.8	0	35		Not estimable
17	29	0	137	32	0	38		Not estimable
4	18	33.33	80	29	30.37	40	0.3%	−11.00 [−22.91, 0.91]
11	39.1	17.8	80	46.1	22.2	38	0.6%	−7.00 [−15.06, 1.06]
21	19.8	8.8	20	19.5	11.6	20	0.9%	0.30 [−6.08, 6.68]
7	17.6	6.79	14	17.42	4.54	7	1.4%	0.18 [−4.72, 5.08]
15	21.3	9.5	84	21.3	10.3	21	1.4%	0.00 [−4.85, 4.85]
10	27	11.48	68	27	14.81	55	1.5%	0.00 [−4.77, 4.77]
6	25.6	10.4	40	22.8	11.5	48	1.6%	2.80 [−1.78, 7.38]
16	23.4	10.7	97	22.1	10.5	43	2.2%	1.30 [−2.49, 5.09]
20	18	7.41	61	15	9.63	36	2.3%	3.00 [−0.65, 6.65]
1	20	8.15	37	24	6.67	46	2.7%	−4.00 [−7.26, −0.74]
13	12.1	5.9	102	15.7	8.3	102	5.1%	−3.60 [−5.58, −1.62]
8	14	6	92	14	4.8	70	6.0%	0.00 [−1.66, 1.66]
2	28.7	12.6	3621	32.3	14.1	381	6.6%	−3.60 [−5.07, −2.13]
3	15	4.44	100	14	3.7	45	6.9%	1.00 [−0.39, 2.39]
18/2	17	8.15	1633	16	6.67	130	7.5%	1.00 [−0.21, 2.21]
5	20.5	6.1	142	20.4	3.8	142	7.6%	0.10 [−1.08, 1.28]
18/1	21	9.63	7821	23	9.63	281	7.7%	−2.00 [−3.15, −0.85]
14	14.33	5.23	255	14.37	4.64	205	8.6%	−0.04 [−0.94, 0.86]
18/3	17	8.15	4869	17	7.41	685	9.5%	0.00 [−0.60, 0.60]
19/1	22.57	10	4704	23.84	11	2352	9.7%	−1.27 [−1.80, −0.74]
19/2	21.03	10	6246	21.7	11	3123	9.9%	−0.67 [−1.13, −0.21]
**Total (95% CI)**			**30338**			**7943**	**100.0%**	**−0.65 [−1.28, −0.02]**

Heterogeneity: Tau² = 0.99; Chi² = 72.21, df = 20 (P < 0.00001); I² = 72%
Test for overall effect: Z = 2.03 (P = 0.04)

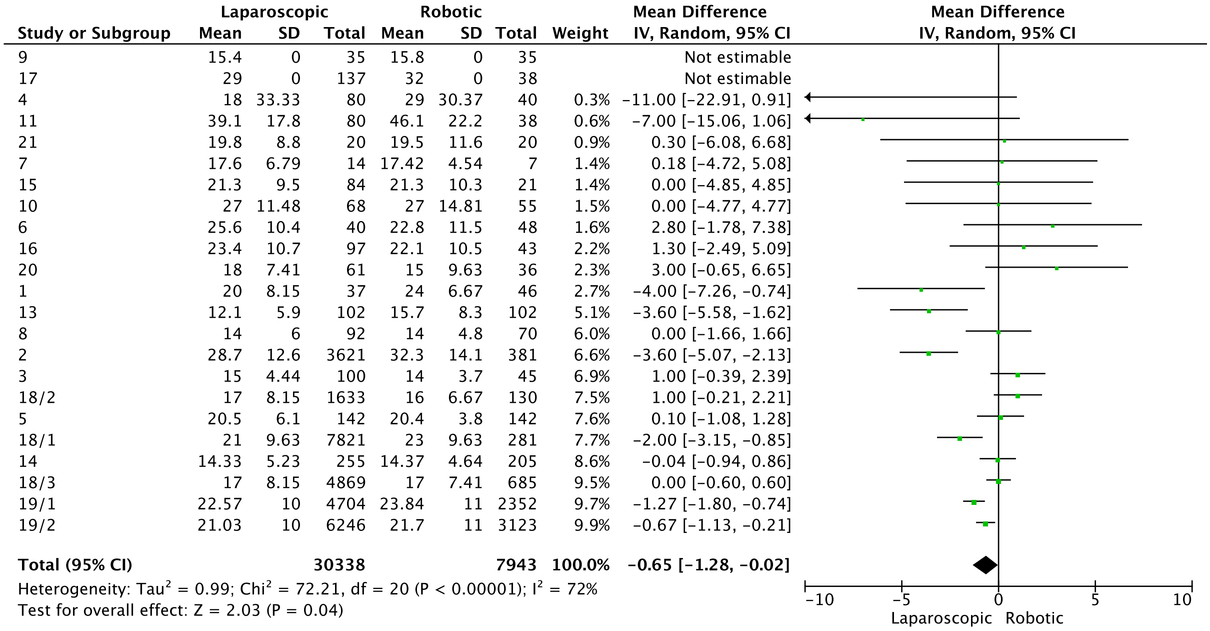

Study or Subgroup	Laparoscopic Events	Laparoscopic Total	Robotic Events	Robotic Total	Weight	Odds Ratio M-H, Fixed, 95% CI
1	2	37	0	46	0.1%	6.55 [0.30, 140.75]
10	4	68	3	55	0.6%	1.08 [0.23, 5.06]
11	0	80	2	38	0.7%	0.09 [0.00, 1.94]
13	4	102	4	102	0.8%	1.00 [0.24, 4.11]
14	17	255	13	205	2.6%	1.05 [0.50, 2.23]
17	18	137	3	38	0.8%	1.76 [0.49, 6.34]
19/1	296	4704	154	2352	37.7%	0.96 [0.78, 1.17]
19/2	358	6246	173	3123	42.6%	1.04 [0.86, 1.25]
2	312	3621	35	381	11.3%	0.93 [0.65, 1.34]
20	3	61	0	36	0.1%	4.37 [0.22, 87.02]
21	0	20	1	20	0.3%	0.32 [0.01, 8.26]
3	1	100	0	45	0.1%	1.37 [0.05, 34.33]
4	4	80	2	40	0.5%	1.00 [0.18, 5.71]
5	2	142	3	142	0.6%	0.66 [0.11, 4.02]
6	2	40	3	48	0.5%	0.79 [0.13, 4.97]
7	0	15	0	7		Not estimable
8	1	92	0	70	0.1%	2.31 [0.09, 57.60]
9	4	35	3	35	0.5%	1.38 [0.28, 6.66]
**Total (95% CI)**		**15835**		**6783**	**100.0%**	**1.00 [0.89, 1.13]**
Total events	1028		399			

Heterogeneity: $Chi^2 = 7.18$, df = 16 (P = 0.97); $I^2 = 0\%$
Test for overall effect: Z = 0.03 (P = 0.98)

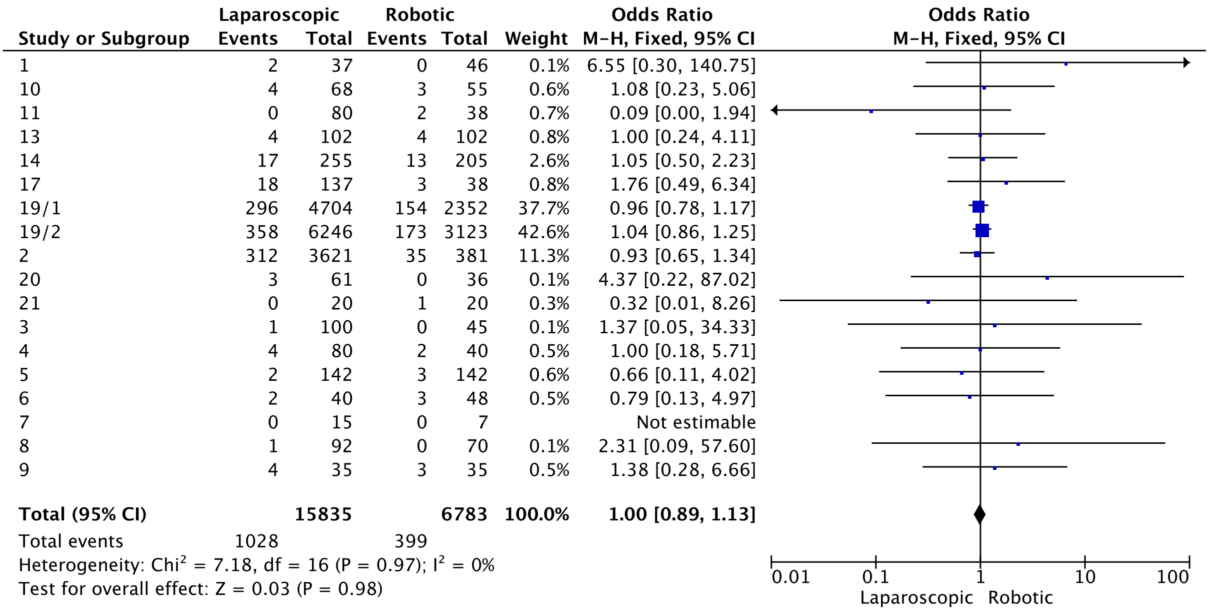

Study or Subgroup	Laparoscopic Events	Laparoscopic Total	Robotic Events	Robotic Total	Weight	Risk Difference M-H, Fixed, 95% CI
11	0	80	1	38	3.3%	-0.03 [-0.09, 0.04]
13	0	102	0	102	6.6%	0.00 [-0.02, 0.02]
14	0	255	0	205	14.6%	0.00 [-0.01, 0.01]
16	0	97	0	43	3.8%	0.00 [-0.03, 0.03]
17	0	137	1	38	3.8%	-0.03 [-0.09, 0.03]
2	322	3621	33	381	44.4%	0.00 [-0.03, 0.03]
4	0	80	1	40	3.4%	-0.03 [-0.08, 0.03]
5	0	142	0	142	9.1%	0.00 [-0.01, 0.01]
6	0	40	0	48	2.8%	0.00 [-0.04, 0.04]
7	0	15	0	7	0.6%	0.00 [-0.19, 0.19]
8	0	92	0	70	5.1%	0.00 [-0.02, 0.02]
9	0	35	0	35	2.3%	0.00 [-0.05, 0.05]
**Total (95% CI)**		**4696**		**1149**	**100.0%**	**-0.00 [-0.02, 0.01]**
Total events	322		36			

Heterogeneity: $Chi^2 = 2.14$, df = 11 (P = 1.00); $I^2 = 0\%$
Test for overall effect: Z = 0.24 (P = 0.81)

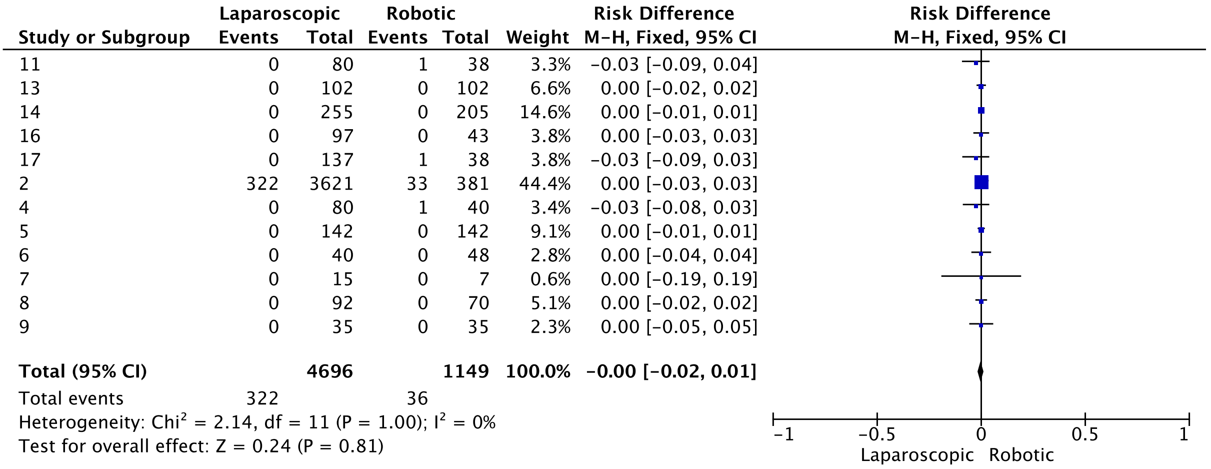

Study or Subgroup	Laparoscopic Mean	Laparoscopic SD	Laparoscopic Total	Robotic Mean	Robotic SD	Robotic Total	Weight	Mean Difference IV, Fixed, 95% CI
11	25.9	12.2	80	22.7	16.8	38	3.5%	3.20 [-2.77, 9.17]
16	11.3	7.3	97	10.8	6.2	43	22.2%	0.50 [-1.85, 2.85]
20	6.2	3.63	61	6.5	3.11	36	66.2%	-0.30 [-1.66, 1.06]
6	14	9.5	40	12.4	9	48	8.1%	1.60 [-2.29, 5.49]
**Total (95% CI)**			**278**			**165**	**100.0%**	**0.15 [-0.96, 1.26]**

Heterogeneity: $Chi^2 = 2.04$, df = 3 (P = 0.56); $I^2 = 0\%$
Test for overall effect: Z = 0.27 (P = 0.79)

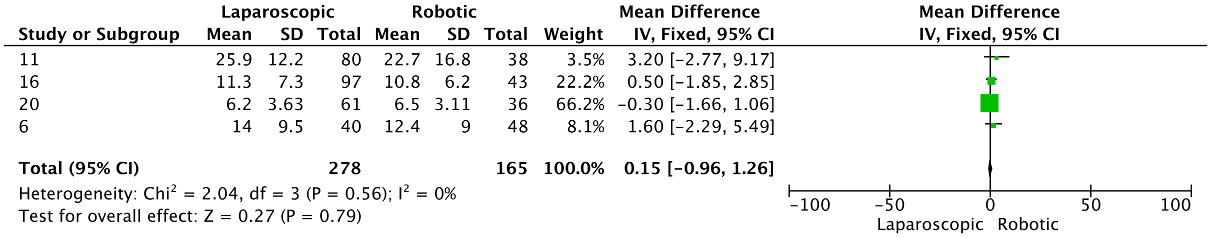

Study or Subgroup	Laparoscopic Events	Laparoscopic Total	Robotic Events	Robotic Total	Weight	Odds Ratio M-H, Random, 95% CI
1	23	37	26	46	1.7%	1.26 [0.52, 3.06]
10	40	68	32	55	2.3%	1.03 [0.50, 2.11]
11	47	80	27	38	1.8%	0.58 [0.25, 1.33]
12	3957	9343	1314	3116	11.0%	1.01 [0.93, 1.09]
13	66	102	71	102	3.2%	0.80 [0.45, 1.44]
14	70	255	123	205	5.3%	0.25 [0.17, 0.37]
15	43	84	9	21	1.4%	1.40 [0.53, 3.67]
16	63	97	12	43	2.0%	4.79 [2.18, 10.51]
17	82	137	20	38	2.3%	1.34 [0.65, 2.76]
18/1	3557	7821	136	281	8.1%	0.89 [0.70, 1.13]
18/2	913	1633	70	130	5.8%	1.09 [0.76, 1.56]
18/3	2886	4869	405	685	9.6%	1.01 [0.86, 1.18]
19/1	2187	4704	1106	2352	10.8%	0.98 [0.89, 1.08]
19/2	3266	6246	1633	3123	11.0%	1.00 [0.92, 1.09]
2	1621	3621	185	381	8.6%	0.86 [0.70, 1.06]
20	38	61	17	36	1.8%	1.85 [0.80, 4.25]
21	13	20	14	20	0.8%	0.80 [0.21, 3.00]
3	41	100	19	45	2.4%	0.95 [0.47, 1.94]
4	37	80	19	40	2.1%	0.95 [0.44, 2.03]
5	79	142	74	142	4.3%	1.15 [0.72, 1.84]
6	28	40	27	48	1.7%	1.81 [0.75, 4.40]
7	8	15	4	7	0.4%	0.86 [0.14, 5.23]
9	20	35	23	35	1.4%	0.70 [0.26, 1.83]
**Total (95% CI)**		**39590**		**10989**	**100.0%**	**0.96 [0.85, 1.08]**
Total events	19085		5366			

Heterogeneity: $Tau^2 = 0.03$; $Chi^2 = 73.59$, df = 22 (P < 0.00001); $I^2 = 70\%$
Test for overall effect: Z = 0.70 (P = 0.48)

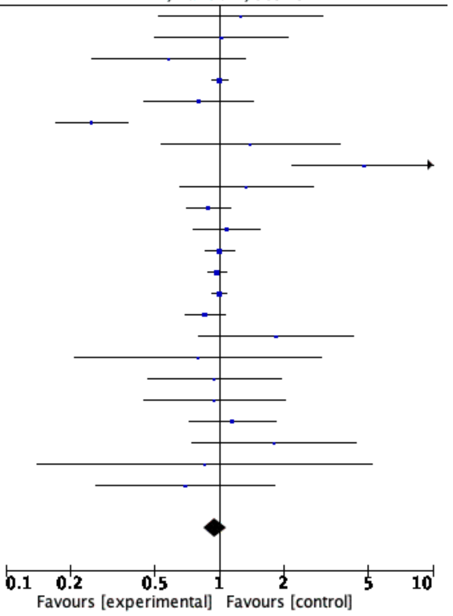

Study or Subgroup	Laparoscopic Events	Laparoscopic Total	Robotic Events	Robotic Total	Weight	Odds Ratio M-H, Random, 95% CI
1	23	37	26	46	1.7%	1.26 [0.52, 3.06]
10	40	68	32	55	2.3%	1.03 [0.50, 2.11]
11	47	80	27	38	1.8%	0.58 [0.25, 1.33]
12	3957	9343	1314	3116	11.0%	1.01 [0.93, 1.09]
13	66	102	71	102	3.2%	0.80 [0.45, 1.44]
14	70	255	123	205	5.3%	0.25 [0.17, 0.37]
15	43	84	9	21	1.4%	1.40 [0.53, 3.67]
16	63	97	12	43	2.0%	4.79 [2.18, 10.51]
17	82	137	20	38	2.3%	1.34 [0.65, 2.76]
18/1	3557	7821	136	281	8.1%	0.89 [0.70, 1.13]
18/2	913	1633	70	130	5.8%	1.09 [0.76, 1.56]
18/3	2886	4869	405	685	9.6%	1.01 [0.86, 1.18]
19/1	2187	4704	1106	2352	10.8%	0.98 [0.89, 1.08]
19/2	3266	6246	1633	3123	11.0%	1.00 [0.92, 1.09]
2	1621	3621	185	381	8.6%	0.86 [0.70, 1.06]
20	38	61	17	36	1.8%	1.85 [0.80, 4.25]
21	13	20	14	20	0.8%	0.80 [0.21, 3.00]
3	41	100	19	45	2.4%	0.95 [0.47, 1.94]
4	37	80	19	40	2.1%	0.95 [0.44, 2.03]
5	79	142	74	142	4.3%	1.15 [0.72, 1.84]
6	28	40	27	48	1.7%	1.81 [0.75, 4.40]
7	8	15	4	7	0.4%	0.86 [0.14, 5.23]
9	20	35	23	35	1.4%	0.70 [0.26, 1.83]
**Total (95% CI)**		**39590**		**10989**	**100.0%**	**0.96 [0.85, 1.08]**
Total events	19085		5366			

Heterogeneity: $Tau^2 = 0.03$; $Chi^2 = 73.59$, df = 22 (P < 0.00001); $I^2 = 70\%$
Test for overall effect: Z = 0.70 (P = 0.48)

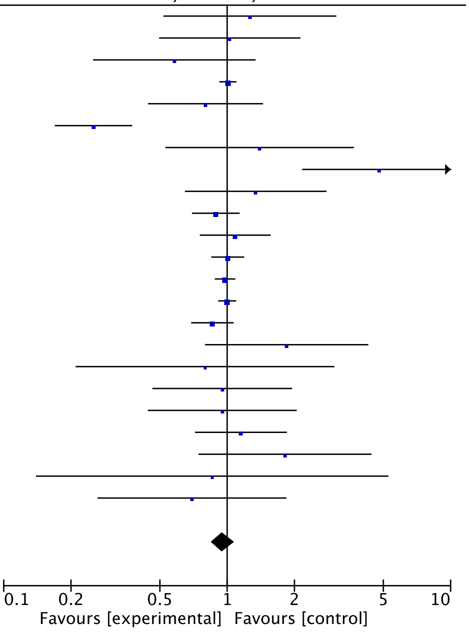

Study or Subgroup	Laparoscopic Mean	Laparoscopic SD	Laparoscopic Total	Robotic Mean	Robotic SD	Robotic Total	Weight	Mean Difference IV, Random, 95% CI
11	44.3	16.9	80	47.4	23.1	38	13.8%	−3.10 [−11.33, 5.13]
21	27.4	10	20	26.6	9.1	20	18.9%	0.80 [−5.13, 6.73]
4	26	8.74	80	32.2	6.67	40	27.5%	−6.20 [−9.02, −3.38]
6	33.7	9.7	40	31.9	8.7	48	24.6%	1.80 [−2.09, 5.69]
7	23.07	8.46	14	23.71	8.13	7	15.3%	−0.64 [−8.12, 6.84]
**Total (95% CI)**			**234**			**153**	**100.0%**	**−1.64 [−5.69, 2.42]**

Heterogeneity: $Tau^2 = 13.50$; $Chi^2 = 12.72$, df = 4 (P = 0.01); $I^2 = 69\%$
Test for overall effect: Z = 0.79 (P = 0.43)

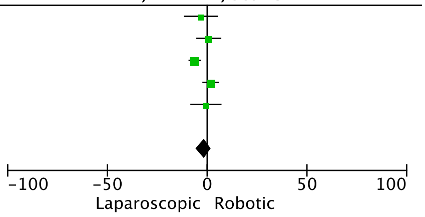

Study or Subgroup	Laparoscopic Events	Laparoscopic Total	Robotic Events	Robotic Total	Weight	Odds Ratio M-H, Fixed, 95% CI
1	12	37	26	46	1.1%	0.37 [0.15, 0.91]
10	23	68	11	55	0.6%	2.04 [0.89, 4.69]
11	33	80	18	38	1.0%	0.78 [0.36, 1.70]
12	2894	9343	954	3116	70.7%	1.02 [0.93, 1.11]
13	35	102	37	102	1.7%	0.92 [0.52, 1.63]
14	87	255	67	205	3.5%	1.07 [0.72, 1.58]
16	37	97	20	43	1.2%	0.71 [0.34, 1.47]
17	55	137	15	38	1.0%	1.03 [0.49, 2.14]
2	1342	3621	142	381	11.6%	0.99 [0.80, 1.23]
20	25	61	10	36	0.5%	1.81 [0.74, 4.40]
3	48	100	23	45	1.2%	0.88 [0.44, 1.79]
4	34	80	15	40	0.8%	1.23 [0.57, 2.68]
5	54	142	55	142	2.4%	0.97 [0.60, 1.57]
6	15	40	12	48	0.5%	1.80 [0.72, 4.49]
7	3	15	1	7	0.1%	1.50 [0.13, 17.67]
8	33	92	31	70	1.6%	0.70 [0.37, 1.33]
9	7	35	6	35	0.3%	1.21 [0.36, 4.04]
**Total (95% CI)**		**14305**		**4447**	**100.0%**	**1.01 [0.94, 1.09]**
Total events	4737		1443			

Heterogeneity: $Chi^2 = 14.14$, df = 16 (P = 0.59); $I^2 = 0\%$

Test for overall effect: Z = 0.25 (P = 0.80)

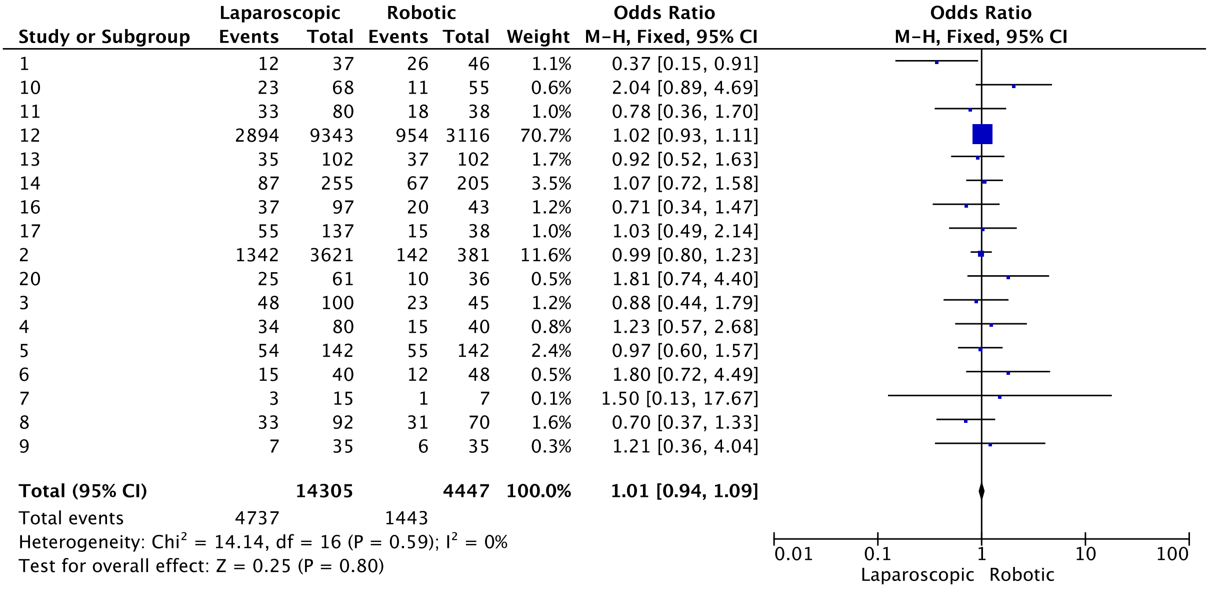

Study or Subgroup	Laparoscopic Mean	Laparoscopic SD	Laparoscopic Total	Robotic Mean	Robotic SD	Robotic Total	Weight	Mean Difference IV, Random, 95% CI
1	7	1.48	37	6	1.48	46	5.2%	1.00 [0.36, 1.64]
10	7	3.33	68	6	1.48	55	4.2%	1.00 [0.12, 1.88]
11	7.9	4	80	7.2	3.1	38	2.7%	0.70 [−0.62, 2.02]
12	6.4	5.1	9343	5.9	6	3116	6.9%	0.50 [0.27, 0.73]
13	8.7	3	102	8.7	4.4	102	3.6%	0.00 [−1.03, 1.03]
14	9.14	3.08	255	9.23	3.37	205	5.4%	−0.09 [−0.69, 0.51]
15	9.3	6.5	84	8.3	1.7	21	2.2%	1.00 [−0.57, 2.57]
16	8.6	3.5	97	7.7	2	43	4.0%	0.90 [−0.02, 1.82]
17	9	2.96	137	7	4.44	38	2.3%	2.00 [0.50, 3.50]
18/1	4	2.96	7821	4	2.22	281	6.8%	0.00 [−0.27, 0.27]
18/2	4	2.96	1663	5	2.96	130	5.7%	−1.00 [−1.53, −0.47]
18/3	4	1.48	4869	4	1.48	685	7.2%	0.00 [−0.12, 0.12]
19/1	4.7	3.8	4704	4	3.4	2352	7.1%	0.70 [0.52, 0.88]
19/2	4.6	3.8	6246	3	3.5	3123	7.1%	1.60 [1.45, 1.75]
20	9	2.22	61	9	2.22	36	4.0%	0.00 [−0.91, 0.91]
21	7.8	3	20	7.2	1.6	20	2.3%	0.60 [−0.89, 2.09]
3	8	5.93	100	7	5.19	45	1.6%	1.00 [−0.91, 2.91]
4	5	3.71	100	6	4.45	40	2.2%	−1.00 [−2.56, 0.56]
5	9	4.7	142	8.7	3.4	142	3.9%	0.30 [−0.65, 1.25]
6	7.4	3.17	40	7.8	2.67	48	2.9%	−0.40 [−1.64, 0.84]
7	7.67	1.23	15	7.71	1.79	7	2.4%	−0.04 [−1.50, 1.42]
8	8.7	4.8	92	7.7	3.1	70	3.0%	1.00 [−0.22, 2.22]
9	8.7	0.1	25	8.3	0.1	25	7.2%	0.40 [0.34, 0.46]
Total (95% CI)			36101			10668	100.0%	0.42 [0.14, 0.69]

Heterogeneity: $Tau^2 = 0.27$; $Chi^2 = 340.09$, df = 22 ($P < 0.00001$); $I^2 = 94\%$

Test for overall effect: Z = 2.97 (P = 0.003)

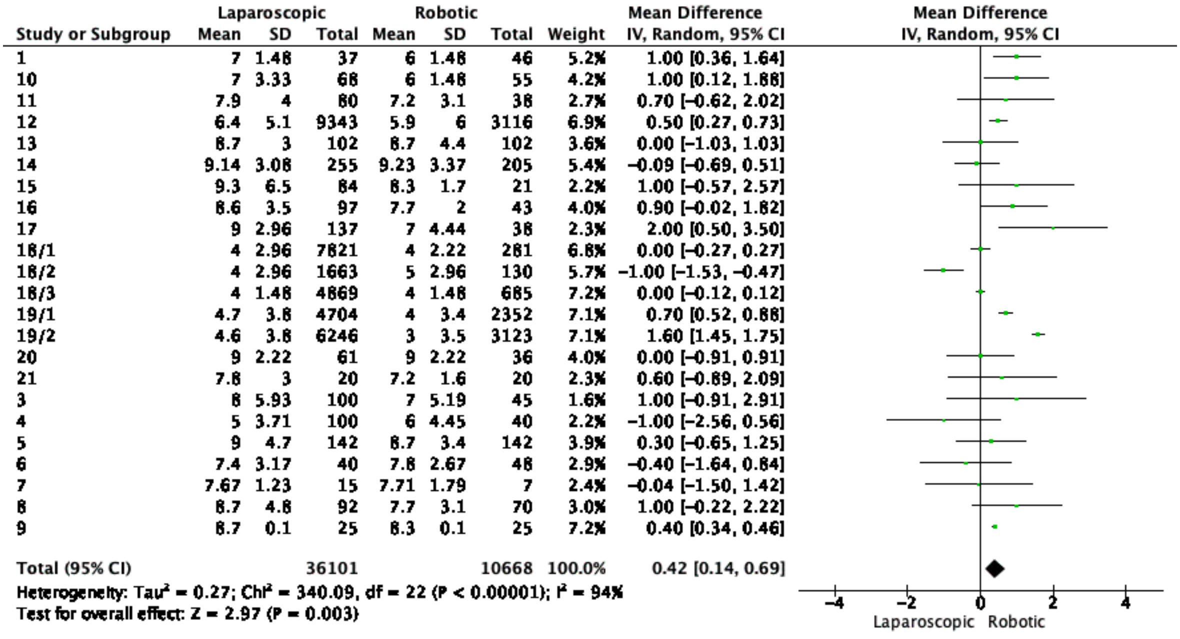

Study or Subgroup	Laparoscopic Mean	Laparoscopic SD	Laparoscopic Total	Robotic Mean	Robotic SD	Robotic Total	Weight	Mean Difference IV, Random, 95% CI
1	20	8.15	37	24	6.67	46	2.7%	−4.00 [−7.26, −0.74]
10	27	11.48	68	27	14.81	55	1.5%	0.00 [−4.77, 4.77]
11	39.1	17.8	80	46.1	22.2	38	0.6%	−7.00 [−15.06, 1.06]
13	12.1	5.9	102	15.7	8.3	102	5.1%	−3.60 [−5.58, −1.62]
14	14.33	5.23	255	14.37	4.64	205	8.6%	−0.04 [−0.94, 0.86]
15	21.3	9.5	84	21.3	10.3	21	1.4%	0.00 [−4.85, 4.85]
16	23.4	10.7	97	22.1	10.5	43	2.2%	1.30 [−2.49, 5.09]
17	29	0	137	32	0	38		Not estimable
18/1	21	9.63	7821	23	9.63	281	7.7%	−2.00 [−3.15, −0.85]
18/2	17	8.15	1633	16	6.67	130	7.5%	1.00 [−0.21, 2.21]
18/3	17	8.15	4869	17	7.41	685	9.5%	0.00 [−0.60, 0.60]
19/1	22.57	10	4704	23.84	11	2352	9.7%	−1.27 [−1.80, −0.74]
19/2	21.03	10	6246	21.7	11	3123	9.9%	−0.67 [−1.13, −0.21]
2	28.7	12.6	3621	32.3	14.1	381	6.6%	−3.60 [−5.07, −2.13]
20	18	7.41	61	15	9.63	36	2.3%	3.00 [−0.65, 6.65]
21	19.8	8.8	20	19.5	11.6	20	0.9%	0.30 [−6.08, 6.68]
3	15	4.44	100	14	3.7	45	6.9%	1.00 [−0.39, 2.39]
4	18	33.33	80	29	30.37	40	0.3%	−11.00 [−22.91, 0.91]
5	20.5	6.1	142	20.4	3.8	142	7.6%	0.10 [−1.08, 1.28]
6	25.6	10.4	40	22.8	11.5	48	1.6%	2.80 [−1.78, 7.38]
7	17.6	6.79	14	17.42	4.54	7	1.4%	0.18 [−4.72, 5.08]
8	14	6	92	14	4.8	70	6.0%	0.00 [−1.66, 1.66]
9	15.4	0	35	15.8	0	35		Not estimable
Total (95% CI)			30338			7943	100.0%	−0.65 [−1.28, −0.02]

Heterogeneity: $Tau^2 = 0.99$; $Chi^2 = 72.21$, df = 20 ($P < 0.00001$); $I^2 = 72\%$
Test for overall effect: Z = 2.03 (P = 0.04)

Mean Difference IV, Random, 95% CI

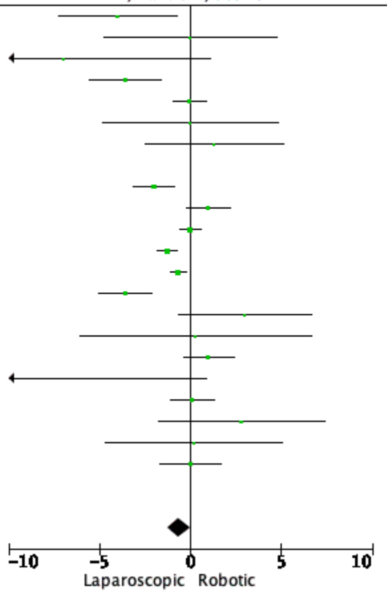

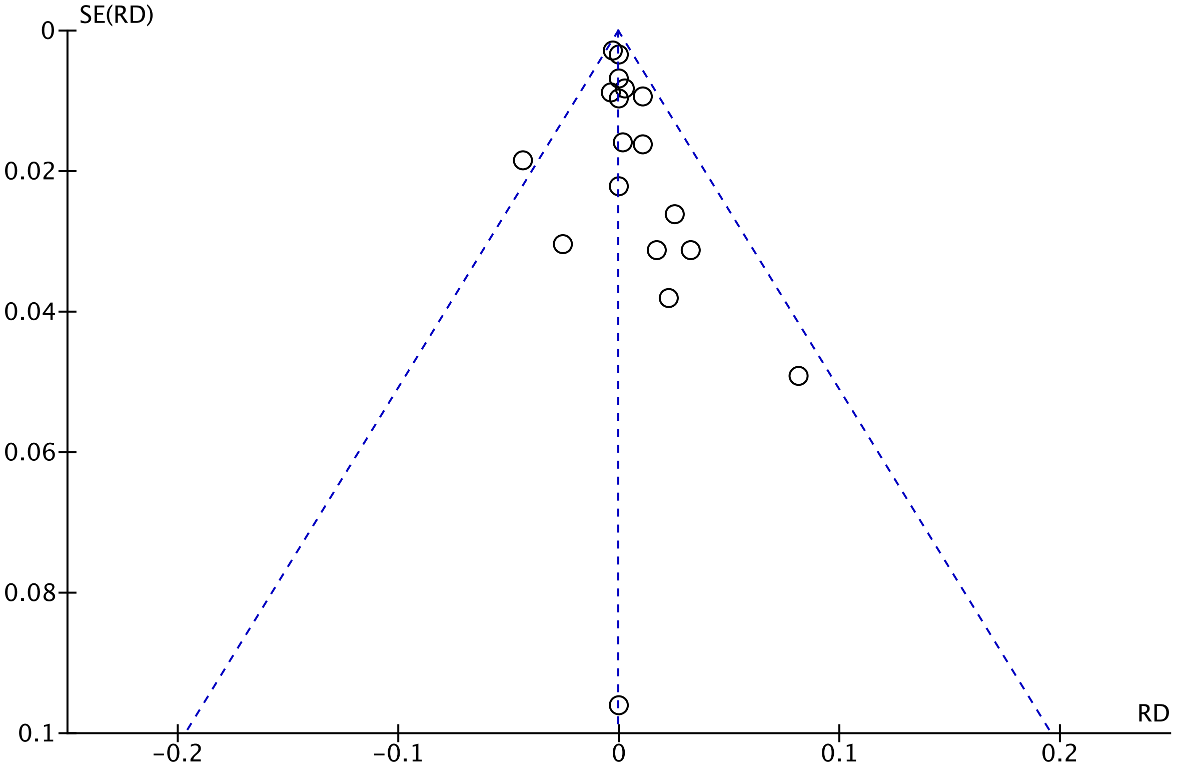

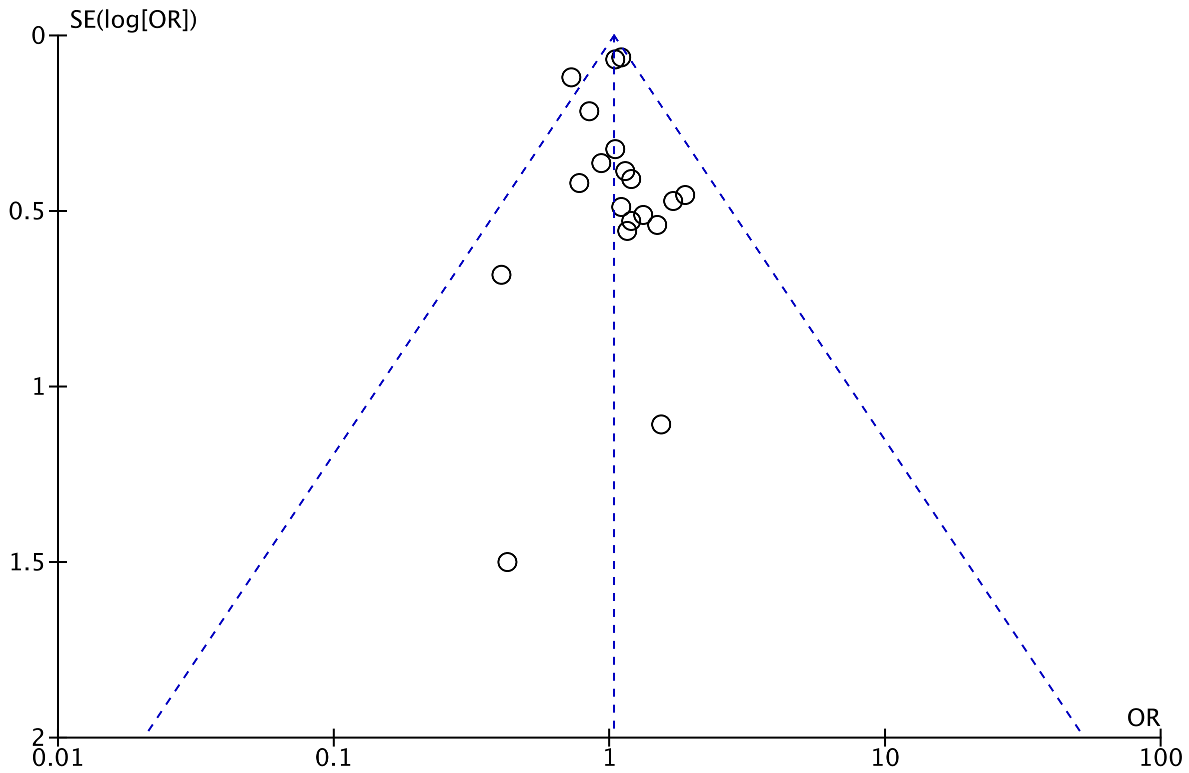

SE(log[OR])
0
0.5
1
1.5
2
OR
0.01
0.1
1
10
100

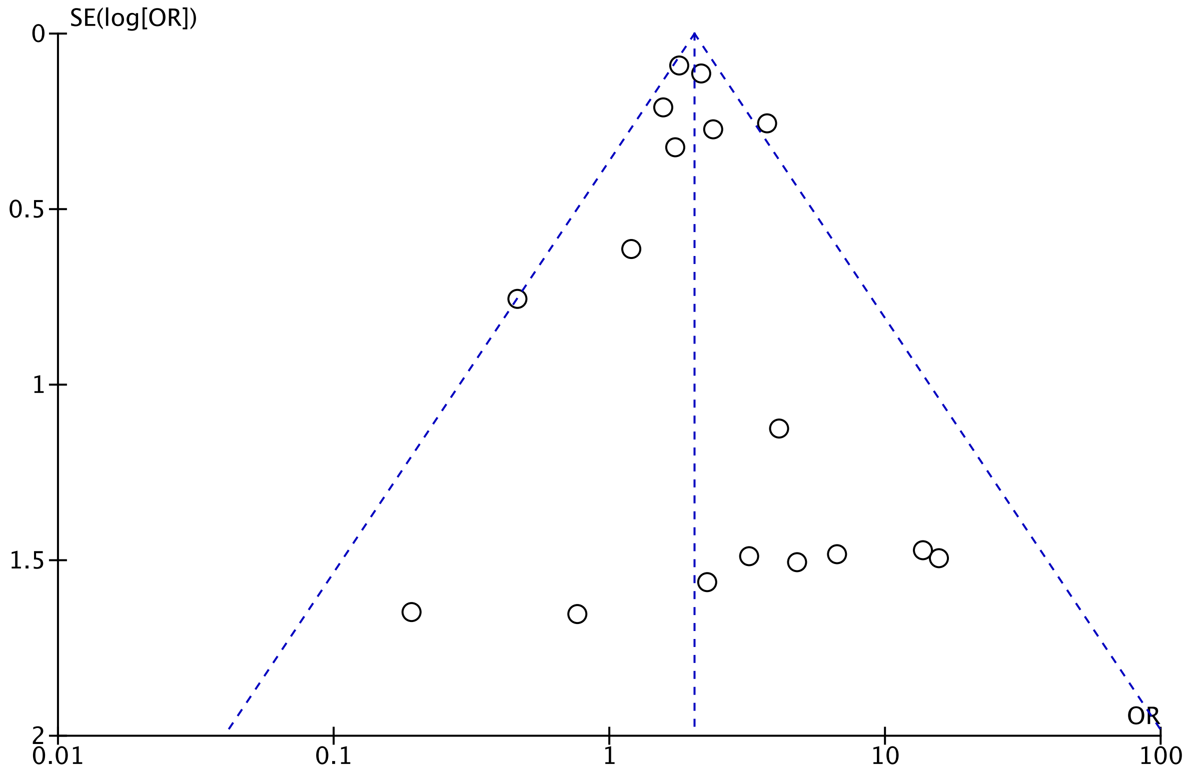

SE(log[OR])
0
0.5
1
1.5
2
OR
0.01
0.1
1
10
100

	Laparoscopic			Robotic				Std. Mean Difference
Study or Subgroup	Mean	SD	Total	Mean	SD	Total	Weight	IV, Random, 95% CI
1	150	9	37	262	16.5	46	4.1%	−8.11 [−9.44, −6.77]
7	104.2	12.03	15	142.22	22.05	7	4.4%	−2.33 [−3.50, −1.15]
20	155	14.17	61	232	13.83	36	4.9%	−5.44 [−6.32, −4.56]
17	238	4.83	137	195	8.67	38	4.9%	7.30 [6.45, 8.15]
10	142	9.25	68	198	11.83	55	5.0%	−5.31 [−6.07, −4.55]
21	165.9	30.2	20	225.2	73	20	5.2%	−1.04 [−1.71, −0.38]
9	179	25	35	243	40	35	5.3%	−1.90 [−2.47, −1.33]
15	208	53	84	185	46	21	5.4%	0.44 [−0.04, 0.92]
11	159.3	56.1	80	325	123.2	38	5.4%	−1.97 [−2.43, −1.51]
4	130	31.67	80	180	28.67	40	5.4%	−1.62 [−2.05, −1.18]
6	254.2	48	40	265.9	52	48	5.4%	−0.23 [−0.65, 0.19]
16	204.1	41.7	97	232.8	46	43	5.5%	−0.66 [−1.03, −0.30]
3	150	37.04	100	155	37.04	45	5.5%	−0.13 [−0.49, 0.22]
8	186.1	43.7	92	141.4	40	70	5.5%	1.06 [0.72, 1.39]
13	168.9	52.8	102	192.9	53.2	102	5.6%	−0.45 [−0.73, −0.17]
5	182.3	33.4	142	200.9	62.1	142	5.6%	−0.37 [−0.61, −0.14]
14	125.85	38.67	255	150.23	43.77	205	5.6%	−0.59 [−0.78, −0.41]
19/1	134	18	4704	183	20.75	2352	5.6%	−2.58 [−2.65, −2.52]
19/2	154	22.25	6246	202	24.75	3123	5.6%	−2.08 [−2.13, −2.02]
Total (95% CI)			12395			6466	100.0%	−1.27 [−1.79, −0.75]

Heterogeneity: $Tau^2 = 1.24$; $Chi^2 = 2164.40$, df = 18 (P < 0.00001); $I^2 = 99\%$
Test for overall effect: Z = 4.81 (P < 0.00001)

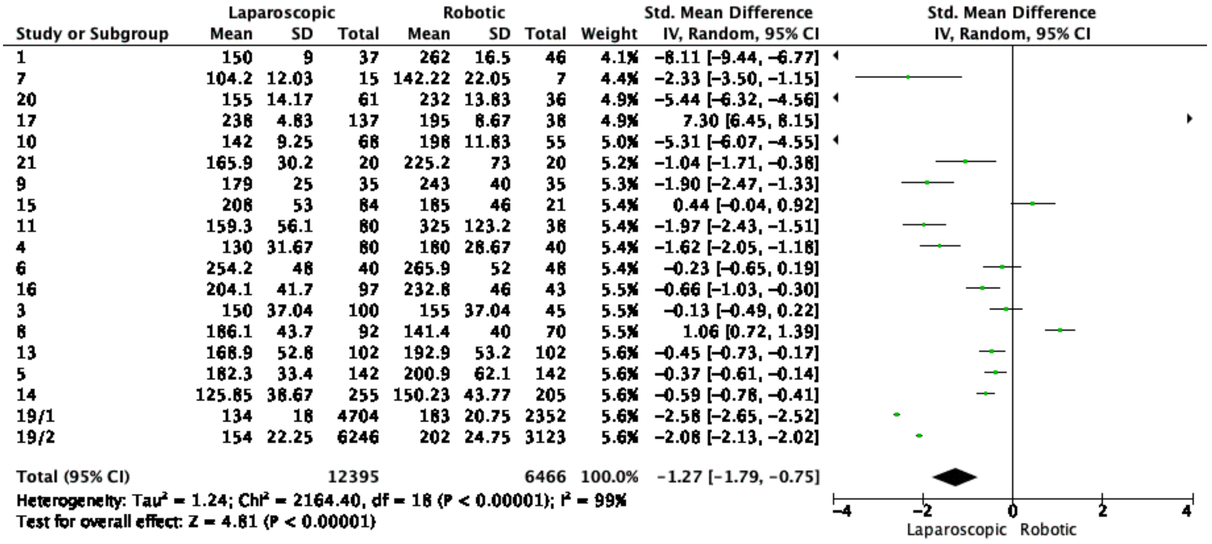

CONVERSION

Classical Meta-Analysis

Fixed and Random Effects

	Q	df	p
Omnibus test of Model Coefficients	12.012	1	< .001
Test of Residual Heterogeneity	1308.755	20	< .001

Note. *p* -values are approximate.
Note. The model was estimated using Restricted ML method.

Coefficients

	Estimate	Standard Error	z	p
intercept	3.030	0.874	3.466	< .001

Note. Wald test.

Residual Heterogeneity Estimates

	Estimate
τ^2	15.102
τ	3.886
I^2 (%)	99.983
H^2	5768.951

Rank correlation test for Funnel plot asymmetry

	Kendall's τ	p
Rank test	-0.029	0.882

Regression test for Funnel plot asymmetry ("Egger's test")		
	**z**	**p**
sei	2.733	0.006

Plot

Funnel Plot

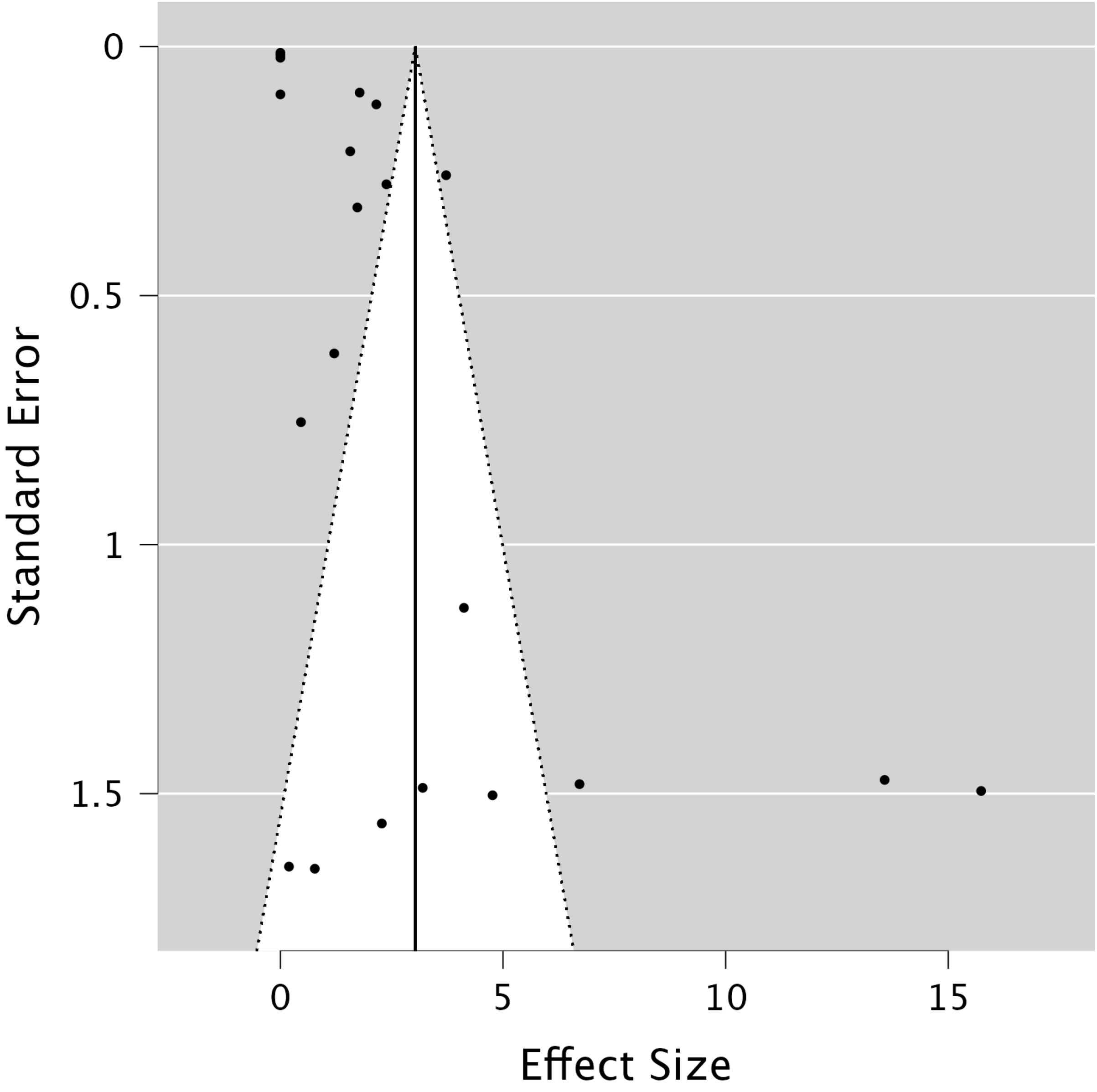

PET-PEESE

Model Tests

Test of Effect

	t	df	p
PET	-1.242	19	0.229

Test of Publication Bias

	t	df	p
PET	4.164	19	< .001

Estimates

Mean Estimates (μ)

						95% Confidence Interval	
	Estimate	Standard Error	t	df	p	Lower	Upper
PET	-0.076	0.061	-1.242	19	0.229	-0.196	0.044
PEESE	0.038	0.069	0.555	19	0.585	-0.097	0.173

Robust Bayesian Meta-Analysis

Summary

Model Summary

	Models	P(M)	P(M|data)	Inclusion BF
Effect	18/36	0.500	0.533	1.140
Heterogeneity	18/36	0.500	1.000	$1.503\times10^{+125}$
Publication bias	32/36	0.500	0.977	41.566

Model Averaged Estimates

			95% CI	
	Mean	Median	Lower	Upper
Effect size (μ)	0.358	0.000	-0.621	1.945
Heterogeneity (τ)	3.295	3.207	2.213	4.850

Note. The estimates are summarized on the none scale (priors were specified on the none scale).

Model Averaged Weights (ω)

p -values interval				95% CI	
Lower	Upper	Mean	Median	Lower	Upper
0.000	0.025	1.000	1.000	1.000	1.000
0.025	0.050	1.000	1.000	1.000	1.000
0.050	0.100	0.997	1.000	1.000	1.000
0.100	0.950	0.994	1.000	1.000	1.000

Model Averaged Weights (ω)

p -values interval				95% CI	
Lower	Upper	Mean	Median	Lower	Upper
0.950	0.975	0.994	1.000	1.000	1.000
0.975	1.000	0.994	1.000	1.000	1.000

Note. (Estimated publication weights omega correspond to one-sided p-values.)

Model Averaged PET-PEESE Estimates

			95% CI	
	Mean	Median	Lower	Upper
PET	2.039	2.474	0.000	4.758
PEESE	0.683	0.000	0.000	3.008

OPERATION TIME

Classical Meta-Analysis

Fixed and Random Effects

	Q	df	p
Omnibus test of Model Coefficients	3.770	1	0.052
Test of Residual Heterogeneity	2164.395	18	< .001

Note. *p* -values are approximate.
Note. The model was estimated using Restricted ML method.

Coefficients

					95% Confidence Interval	
	Estimate	Standard Error	z	p	Lower	Upper
intercept	-1.353	0.697	-1.942	0.052	-2.719	0.013

Note. Wald test.

Residual Heterogeneity Estimates

		95% Confidence Interval	
	Estimate	Lower	Upper
τ^2	9.127	5.190	20.562
τ	3.021	2.278	4.535
I^2 (%)	99.886	99.799	99.949
H^2	875.627	498.376	1971.493

Rank correlation test for Funnel plot asymmetry

	Kendall's τ	p
Rank test	-0.310	0.068

Regression test for Funnel plot asymmetry ("Egger's test")

	z	p
sei	-1.423	0.155

Plot

Funnel Plot

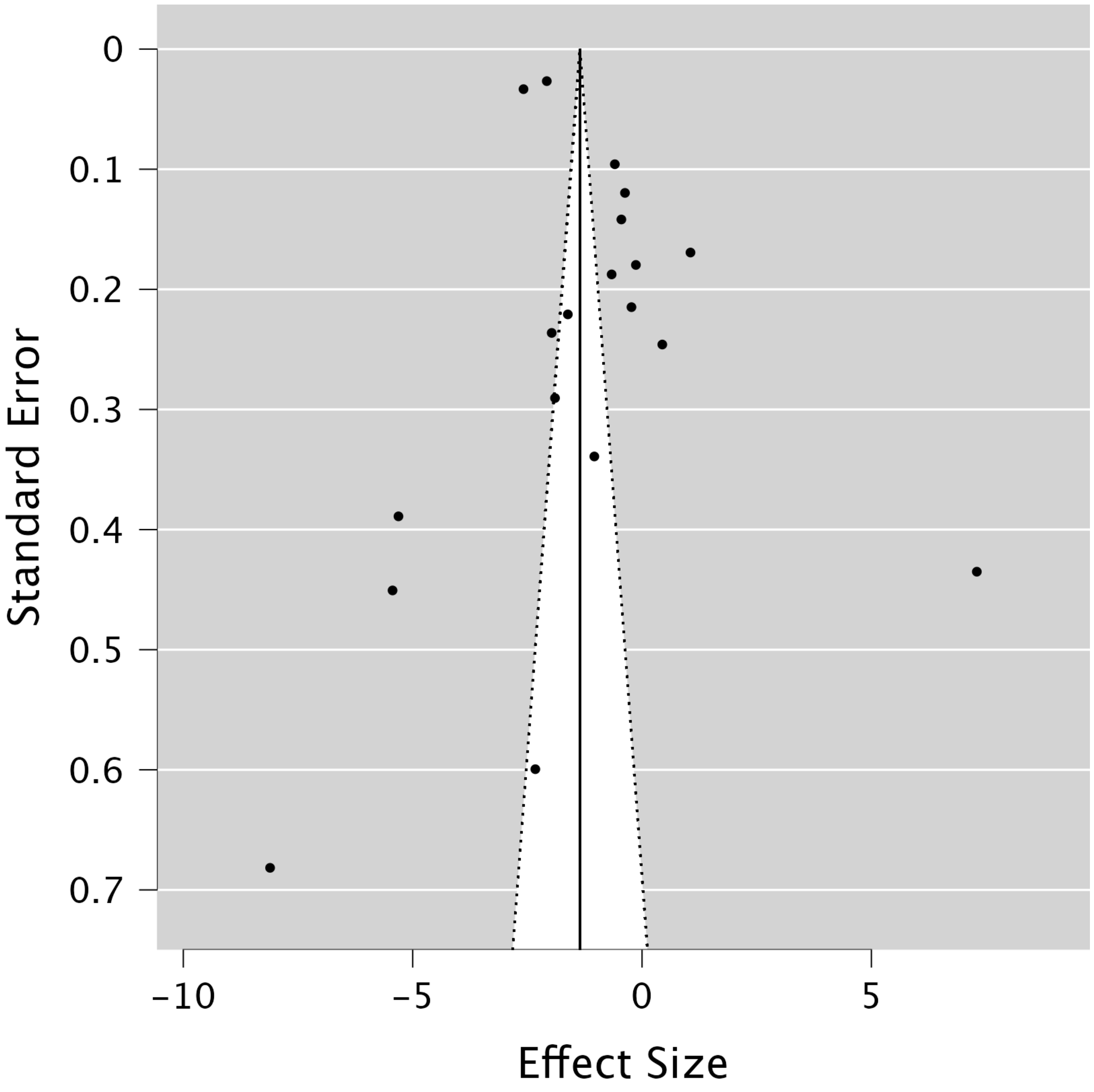

PET-PEESE

Model Tests

Test of Effect

	t	df	p
PET	-9.492	17	< .001

Test of Publication Bias

	t	df	p
PET	2.160	17	0.045

Estimates

Mean Estimates (μ)

						95% Confidence Interval	
	Estimate	Standard Error	t	df	p	Lower	Upper
PET	-2.333	0.246	-9.492	17	< .001	-2.815	-1.851
PEESE	-2.044	0.219	-9.341	17	< .001	-2.473	-1.615

Robust Bayesian Meta-Analysis

Summary

Model Summary

	Models	P(M)	P(M|data)	Inclusion BF
Effect	18/36	0.500	0.483	0.935
Heterogeneity	18/36	0.500	1.000	∞
Publication bias	32/36	0.500	0.826	4.731

Model Averaged Estimates

			95% CI	
	Mean	Median	Lower	Upper
Effect size (μ)	-0.191	0.000	-1.580	0.916
Heterogeneity (τ)	3.046	2.976	2.163	4.357

Note. The estimates are summarized on the none scale (priors were specified on the none scale).

Model Averaged Weights (ω)

p -values interval				95% CI	
Lower	Upper	Mean	Median	Lower	Upper
0.000	0.025	1.000	1.000	1.000	1.000
0.025	0.050	0.997	1.000	1.000	1.000
0.050	0.100	0.856	1.000	0.304	1.000

Model Averaged Weights (ω)

p -values interval				95% CI	
Lower	Upper	Mean	Median	Lower	Upper
0.100	0.950	0.727	1.000	0.136	1.000
0.950	0.975	0.729	1.000	0.136	1.000
0.975	1.000	0.731	1.000	0.136	1.000

Note. (Estimated publication weights omega correspond to one-sided p-values.)

Model Averaged PET-PEESE Estimates

			95% CI	
	Mean	Median	Lower	Upper
PET	0.347	0.000	0.000	4.556
PEESE	2.410	0.000	0.000	14.579
